# Portland Versus Alkaline Cement: Continuity or Clean Break: “A Key Decision for Global Sustainability”

**DOI:** 10.3389/fchem.2021.705475

**Published:** 2021-10-11

**Authors:** A. Palomo, O. Maltseva, I. Garcia-Lodeiro, A. Fernández-Jiménez

**Affiliations:** Eduardo Torroja Institute for Construction Science, IETcc-CSIC, Madrid, Spain

**Keywords:** Alkali Activated Binders, geopolymers, precursor processing, low CO_2_ footprint, activators

## Abstract

This review undertakes rigorous analysis of much of the copious literature available to the scientific community on the use of alkali-activated binders (AABs) in construction. The authors’ main intention is to categorically refute arguments of that part of the scientific community underestimating or even dismissing the actual potential of AABs as alternatives to Portland cement (PC). The main premise invoked in support of those arguments is a presumed lack of material resources for precursors that would make AAB industrial-scale production unfeasible anywhere on the planet (a substantial number of scientific papers show that the raw materials required for AAB manufacture are in abundance worldwide). The review also analyses the role of alkaline activators in the chemistry of AABs; it is important to clarify and highlight that alkaline activators are not, by any means, confined to the two synthetic products (caustic soda and waterglass) mostly employed by researchers; other sustainable and efficient products are widely available. Finally, the review deals with the versatility of AAB production processes. The technologies required for the large scale manufacturing of AABs are mostly already in place in PC factories; actually no huge investment is required to transform a PC plant in a AAB factory; and quality and compositional uniformity of Alkaline Cements (binders produced through an industrial process) would be guaranteed. The last conclusions extracted from this review-paper are related with: i) the low carbon footprint of one-part AABs and ii) the urgent need of exploring standardization formulas allowing the commercial development of (sustainable) binders different from PC.

**GRAPHICAL ABSTRACT:**
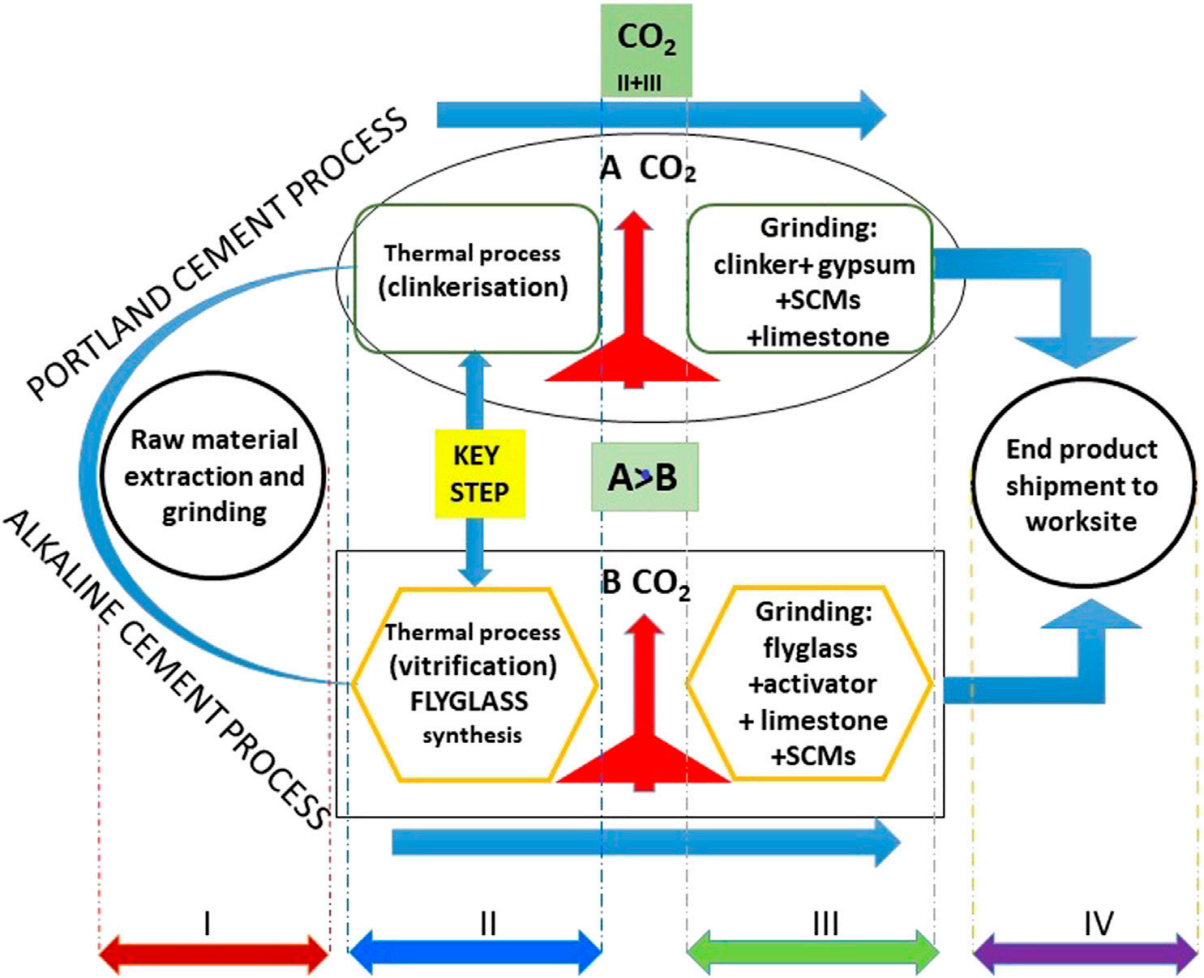


## Introduction

According to reports issued by the United Nations Climate Change Conference (COP 25) held at Madrid, Spain in December 2019, the ice cap in Greenland is melting seven times more quickly than in the nineteen nineties and one-fourth of the world’s population is at risk of water shortage in the near future. NASA data also reveal that the five warmest years on record for the planet as a whole occurred since 2010. The United Nations Secretary General periodically reminds the world of the need to respond speedily to the threat of climate change, for further delaying decisions entails assuming many risks, incurring higher costs and forgoing opportunities to modernize the society. The primary COP 25 agreement was to lay the grounds for the international community to present more ambitious plans for tackling climate change in 2020 than in 2015, in line with societal demands and scientific recommendations.

The 2015 Paris Agreement (European Commission, 2015) established an action plan to prevent the planet’s temperature from rising by more than 2°C. That target was deemed reachable only if greenhouse gas (GHG) emissions could be significantly reduced. It was therefore agreed to establish fairly urgent measures in the energy industry and further the circular economy in all manufacturing sectors.

On the grounds of the size of its environmental footprint and enormous economic and social significance in the vast majority of the world’s countries, construction has been singled out by many authors and institutions as a strategic industry where sustainability policies should be applied without delay. Insight into the importance of construction in tackling climate change can be gleaned from the numbers: buildings (construction and operation) consume 36% of all the energy produced worldwide and account for 39% of global CO_2_ emissions (Abergel et al., 2018); and the industries that produce the main building materials, cement and steel, jointly emit 12% of the world’s CO_2_ (Favier et al., 2018).

The primary problem facing construction is that it is largely patterned on traditional, energy-intensive production models characterised by high GHG emissions. Such models are consequently in pressing need of modernization. In the near future, the literature on sustainable construction (Gan et al., 2015) should be one of the keys to worldwide sustainable development.

As far as construction materials are concerned, more concrete is consumed by humanity than any other commodity except water (IEA, 2015). The Bill and Melinda Gates Foundation recently published data on the magnitude of worldwide concrete consumption. According to that foundation, in the next 40 years (2020–2060) the area occupied by the planet’s building and infrastructure assets will grow by 2 Tft2. That is tantamount to saying that in the 40 years to come, the planet’s inhabitants will build a city the size of New York every 30 days (Gates, 2019). Despite those figures, concrete production will in all likelihood not suffice to meet the needs of a growing worldwide population (Barbiere, 2017).

Portland cement (PC), the main component in concrete, converts the plastic mass of aggregate and water into a solid, compact and mechanically sound matrix. As it normally comprises 10–15% of concrete mass, worldwide output will necessarily have to be stepped up to produce the vast volumes of concrete to be consumed in future. Presently estimated to be around 4.5 Gt/year (CEMBUREAU, 2016), global PC output will exceed 5.0 Gt/year in the next 30 years according to some forecasts (IEA, 2017). Whilst developing countries will consume the largest volumes of cement in future, developed nations will also need to repair and restore existing infrastructures and housing stocks, for PC deteriorates with time. One World Bank report estimates that it will cost 6.1% of world GDP in 2015 to repair and maintain all the planet’s infrastructures (Ruiz-Nunez and Wei, 2015).

Excess of PC consumption is a serious problem since PC manufacture is energy-intensive and it is readily understandable, then, that given the huge volumes of concrete consumed, cement today accounts for a substantial share (8-10%) of global CO_2_ emissions (Olivier et al., 2015; Talaei et al., 2019). If the whole cement industry existing in the planet could be installed in one only extensive island, it would rank third after China and the US in total GHG emissions.

**FIGURE 1:**
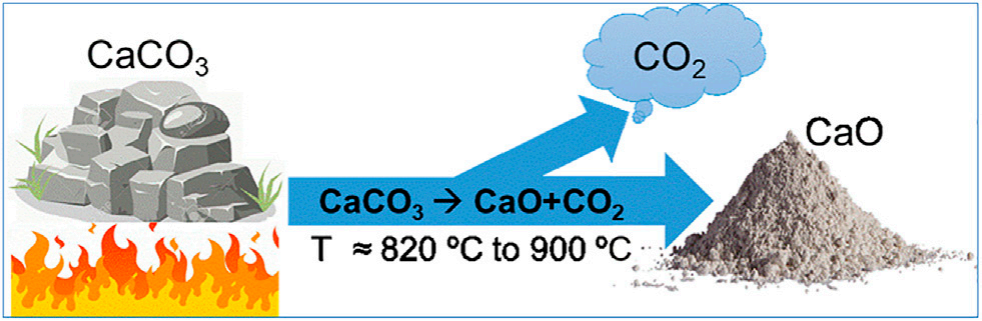
Limestone conversion to CaO.

In the decades to come cement output urgently needed to provide housing and infrastructure for human populations deprived of such assets clashes with the no less urgent need to lower CO_2_ emissions. The mutual exclusivity of those two priorities stems from the objective fact that 60% of CO_2_ emissions associated with PC production is inevitable for it is inherent in the conversion of limestone to CaO (Figure 1). The resulting urgent need to implement low carbon alternatives to PC is a scientific-technical challenge requiring immediate attention, in light of the significant environmental implications involved.

A number of local, regional and international cement manufacturers and their associations have contended that the industry is firmly committed to tackling climate change and openly asserted that the goal is to be in line with the Paris Agreement’s 2°C scenario, by reducing its gross CO_2_ emissions by 30% by 2030 for cement and 40% down the value chain (Cembureau, 2020, 2050 Carbon neutral Roadmap). To reach that aim, they have designed their strategies around lowering both clinker production and Portland cement use (UN Environment et al., 2018) but also around the development of a pan-European CO_2_ transportation and storage network. Actually, carbon capture, use and storage will account for 42% of the CO_2_ emissions reduction in the sector (Cembureau, 2020, 2050 Carbon neutral Roadmap). In other words, the cement industry is relying on very costly and scantly tested technologies such as carbon capture to solve more than 40% of the problem (John et al., 2019) (a problem, by the way, affecting all life on the planet).

Albert Einstein is quoted as saying ”do not pretend that things will change if we always do the same”. That reflection is a warning that persisting in the same working procedures and production models will neither bring change nor solve problems. In short, contrary to Einstein’s recommendation, the cement industry’s plans seem to leave little room for the idea of identifying and developing a new generation of processes and materials. Rather, the sector appears to persist in its refusal to confront the radical change involved in disruptive materials science and technology (Hutchinson, 2016). That attitude persists even though many scientists believe that the cement industry already has a great sketch of sustainable binder, described in detail in the literature but unimplemented for want of construction industry and governmental support and acknowledgement. Many members of the scientific community contend that rethinking the idea of cement is not only an urgent environmental need (adopting a responsible attitude toward the planet’s inhabitants), but a feasible short-term task that would enable other binders to compete on a level building materials playing field, and might guarantee carbon neutrality in a few years.

Over 2000 years ago the Romans manufactured mortars and concretes with clinker-free cements that have passed the test of time and proven to perform extraordinarily well in service (Jackson et al., 2017). The absence, 2000 years later, of any viable cement other than PC cannot be credibly defended.

## Motivation, Objectives, and Originality

A detailed analysis of the abundant literature published during the 21st century reveals that the scientific community working around *sustainable binders for construction* is clearly fragmented and divided in their opinions on the best way to tackle the problem of CO_2_ emissions linked to the cement manufacturing. In a simplified way, it can be said that there exists an extensive “dominant” group of scientists (representing the continuity and supported by the cement industry) whose arguments and work lines are based on the uninterrupted and inexhaustible advance around the knowledge of Portland cement (new crystallochemical details on clinker phases, new evidences on the hydration mechanisms, advances in modelling, durability tests, etc.,); and on the other hand there is a second group of scientists, mainly made up of young scientists, for whom the future implies a break with the past, which in turn is symbolized by the need to decisively promote the development of other binders different to Portland cement (especially Geopolymers). Both groups barely permute positions and barely share common objectives and work criteria, which in real terms mean a mutual disdain (each group shamelessly dispenses with the teachings that the other group might contribute). In this review-paper the argumentation by the dominant group of scientists against the industrial development of results generated by the Geopolymer group are analyzed. Additionally the deficiencies and vices characterizing most of research lines of the second group are also indicated.

Summarizing, this literature review was consequently inspired by a desire to furnish a sound tool for scientific-technical discussion among members of the scientific community who deem diligent progress toward radical change in the cement industry’s environmental strategies to be a priority; and to afford policy-makers a series of valid arguments (endorsed by over 200 scientific publications) with which to pilot this segment of the construction sector toward much more demanding environmental practice than presently in place.

A fuller understanding of the authors’ motivations (the need for an unbiased review on Alkali Activated Binders literature identifying those key lines of work to be intensified, reinforced and prioritized in the near future in order to boost a necessary quick convergence between these binders and the technological reality of global cement production), can be gleaned from an analysis of the following statement: in an interview with World Cement Association President Song Zhiping published in June 2019 by CW Group News, the Chinese entrepreneur contended that ‘no alternative products up to now can effectively replace cement’ (Mr Song’s failure to mention alternative alkaline cements is surprising, given that China, his country of origin, leads the world in the number of scientific-technical papers on alkaline cements). Unfortunately, that statement might be interpreted as an excuse presented to international authorities to justify the industry’s ongoing adherence to traditional strategies. In fact, the message conveyed is: In the absence of viable alternatives to PC, the authorities should work in a scenario that lightens the political pressures on PC producers to attend strict environmental demands.

Driven essentially by that motivation, the authors addressed the primary objective of this article: to show that the scientific-technical certainty about alkaline cements (materialized in the literature on which their technological potential rests) is much fuller, diverse and conclusive than contended in analyses recently published in highly reputed journals (Scrivener et al., 2018; Miller et al., 2018). Such articles not only underestimate the potential of AAB to compete for building material market share in very short order, but also seem to question the credibility of the solutions put forward day after day by a significant part of the scientific community seeking to mitigate the severe environmental problem associated with cement manufacture. That goal is pursued here by critically analyzing a very wide selection of papers to establish: 1) that the raw materials needed to produce AAB are not confined to a series of waste products (materials that would nonetheless contribute to instituting a circular economy), but rather are in abundant supply everywhere on the Earth’s crust; 2) that caustic soda and waterglass, both costly, synthetic, high carbon products, are not the only alkaline activators at hand, for the existence of a broad spectrum of effective and competitive natural products ensures ready AAB manufacture anywhere, worldwide; and 3) that given their diversity and versatility, AAB production processes can draw from technologies that are already in place, call for no huge investment, ensure end product uniformity (and therefore quality) and are characterized by a much lighter environmental impact than generated by PC manufacture.

**FIGURE 2:**
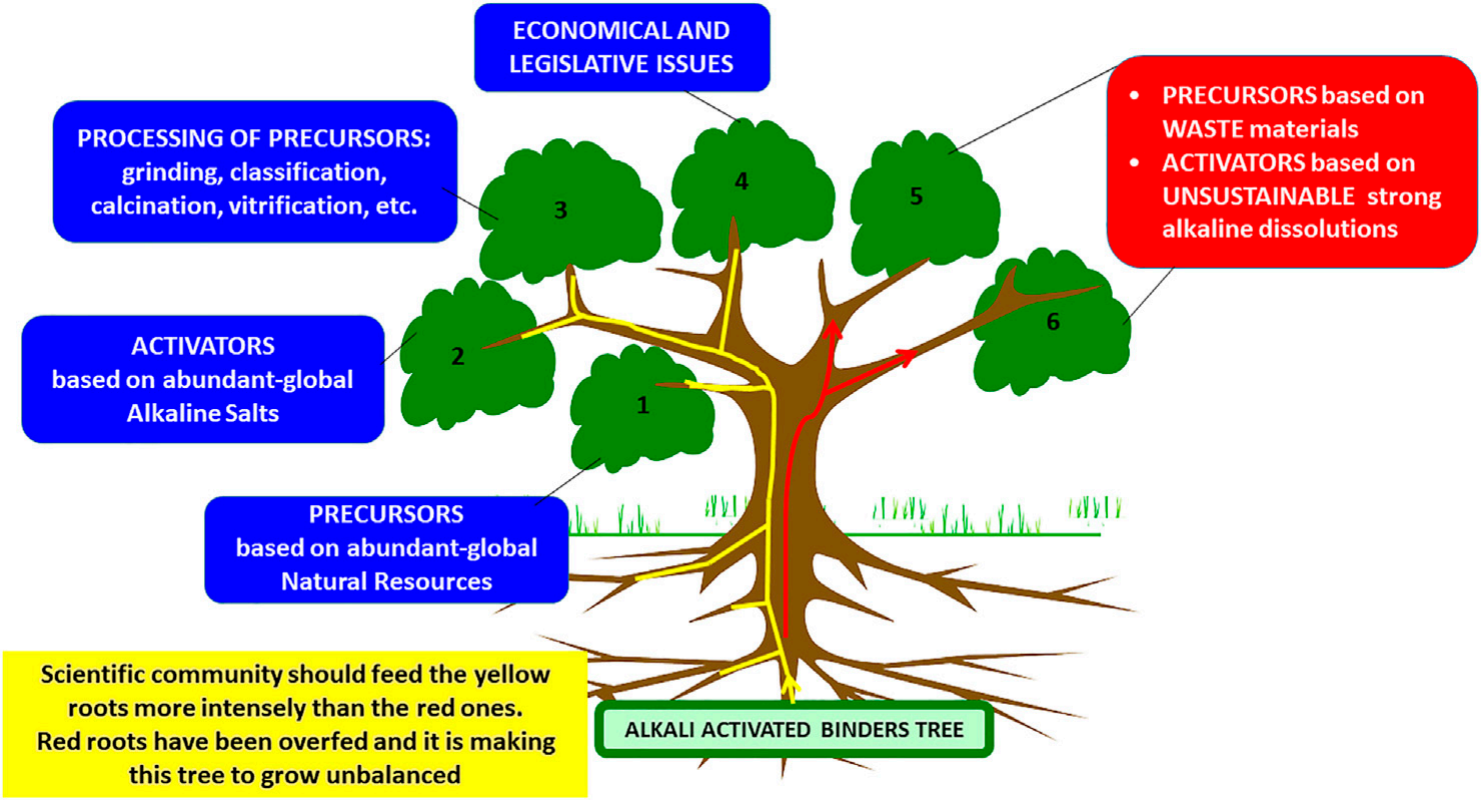
Geopolymer tree. Priority research lines (branches 1, 2, 3 and 4). The tree is also pointing out those research lines (branches 5 and 6) wich are being exhaustively repeated by a huge part of the scientific community (authors of this paper do not reject these lines of work but we believe that the others must be prioritized. Branches 5 and 6 need a period of reflection).

The aforementioned motivation and objective are what differentiate this literature review on AABs from other past exercises (including those contributed to by the present authors, in some cases the ones most frequently cited). That does not mean, however, that the present article fails to explore the recent literature. It does in fact, with the concomitant update of data on object of debate (and the reassertion of the validity of the authors’ scientific criteria) as well as the identification of the most significant gaps in the understanding of sustainable building materials. Actually Figure 2 is a symbolic tree identifying the research lines which should be enhanced in the near future by the Geopolymer scientific community in order to make feasible the prompt presence of AAB in the building materials market (research lines summarized in branches 1, 2, 3, and 4). The tree is also pointing out those research lines (branches 5 and 6) which are being exhaustively repeated by a huge part of the scientific community (authors of this paper do not reject these lines of work but we believe that others should be prioritized).

## Alkali Activated Binders (AABs)*

Alkali activated binders comprise a family of materials (chemically and mineralogically unrelated to Portland cement) generally consisting in two essential components: a cementitious precursor and a chemical additive or alkaline activator. The literature identifies a broad spectrum of industrial, mining and agroforestry by-products or waste and a series of aluminosilicate minerals as precursors (Fernández-Jiménez et al., 2005a; Provis et al., 2015; Van Deventer, 2017; Rivera et al., 2020), whilst caustic alkalis and/or alkaline silicates are frequently used as activators (Provis and Van Deventer, 2014a; Hamidi et al., 2016; Atabey et al., 2020; Garcia-Lodeiro et al., 2020; Zhang et al., 2020). Building on that general formulation, the scientific community has developed a wide variety of materials generically termed AABs (Alkali Activated Binders) or geopolymers. Two main families of AABs can be defined: A) high and B) low Ca content materials. Hybrid cements comprise a third family, consisting in different combinations of A) and B) (Shi et al., 2011; Palomo et al., 2019).

The mechanisms governing the chemical reactions between precursors and alkaline activators differ with family. As a rule in model A), which includes blast furnace slag (Bernal et al., 2014), the main reaction product is a C-(A)-S-H gel (denominated further to the standard chemistry of cement terminology used here (Richardson and Taylor, 2018), similar to the C-S-H gel obtained during PC hydration. In model B), comprising metakaolin or type F fly ash precursors (Palomo et al., 1999; Fernandez-Jimenez et al., 2008; Garg and Skibsted, 2019), the main reaction product is a M-A-S-H gel (M = alkaline cation) (Duxon et al., 2007a), which is attributed the same or higher mechanical performance than C-S-H (Lyngdoh et al., 2019). Overall, AABs comprise a large family of materials characterised by: 1) NO need for clinker; and 2) the need for alkaline activators.

*Even if some researchers consider that the terms “Geopolymer” and “Alkali Activated Binders” do embrace different chemical concepts, in this paper authors have decided to indistinctly use both terms in order not to divert the attention of readers from the main objetives of the paper.

According to the SCOPUS database, over 4,500 papers were published on geopolymers + alkali-activated materials in 2018–2020. Such unprecedented and growing interest in AABs on the part of the scientific community stands as proof that the existing scientific knowledge suffices to establish the many excellent characteristics featured by this family of cements. The pages below contain a series of arguments and discussions intended to clarify some of the widespread misunderstandings around AABs.

### Non-waste Raw Materials apt for Alkaline Activation. Setting the Record Straight

Much of the literature on AABs is recurrent and attests to a certain tendency by certain members of this part of the scientific community to cling to a number of outdated dogmas and therefore to their insufficient contact with cement industry realities. For instance, with the exception of papers dealing with metakaolin (of which there are many, briefly referred to below), 80–90% of the scientific and technical articles on the alkaline activation of aluminosilicates routinely deploy industrial (primarily fly ash and slag), agroforestry, mining or similar waste as AAB precursors. In other words, the term waste would appear to be inevitably associated with the production of alkaline cements (Shi et al., 2007; Bernal et al., 2016; Azevedo et al., 2020; Kioupis et al., 2020; Rivera et al., 2020).

In general terms, any material that has a certain amount of reactive silica and alumina (preferably with Si/Al ratios >1.5) and with certain (preferably high) degree of structural disorder (amorphous or glassy materials) can be used as a precursor in the preparation of AABs. Materials whose reactivity can be modified/increased by initial thermal, mechanical or chemical pre-treatment, can also be used as a precursors. The final yield will depend on the thermodynamic driving force for the desired reaction to happen, and on the dissolution kinetics in alkaline media, which must be fast enough to take place in technologically feasible times. More specific information on the different types of precursors can be found in the references (Ben Haha et al., 2011b; Palomo et al., 2014; Abdullah et al., 2020; Khalifa et al., 2020; Mejía-Arcila et al., 2020; Rivera et al., 2020; Cheah et al., 2021).

The reuse of waste in any human (industrial, agricultural) activity is obviously recommendable and today a practice on the rise the world over in keeping with circular economy principles (which are also logically applicable to the cement and concrete industry). It is no less obvious, however, that this mature and well organized industry should categorically refuse to allow the manufacture of the world’s primary building material (which should be deemed a prime necessity) to depend entirely on the supply of waste products generated by other sectors unrelated to cement or construction. Such a refusal would be justified less by the fact that the vast chemical, physical and mineralogical variability of any waste and its uneven geographic availability would prevent cement production to meet certain minimum quality standards than by the acknowledgement that universal output could not be ensured. Indeed, the present recurrence of data presenting waste activation as intrinsic to alkaline cements (as if those data comprised the sole scientific-technical information on alkali-activatable precursors) portrays them in an adverse light capitalized on by the industry to argue against their use. Such arguments feed mistrust on the large-scale viability of AABs, call the economic cost-effectiveness of the respective cements into question and even cast doubts on their low carbon credentials. Oral and written discussion of precursors apt for alkaline activation (i.e., that set and harden in alkaline media to ultimately form compact, mechanically strong and durable matrices) in fact often contend that ‘these materials are the same products used to replace clinker in blends, substances whose limited availability is well known’ (UN Environment et al., 2018). The message conveyed to anyone possibly considering the possibility of developing AABs industrially and commercially is that such cements could never be an alternative to PC because a steady supply of precursors cannot be guaranteed over time.

For those reasons the focus in this *Non-waste Raw Materials apt for Alkaline Activation. Setting the Record Straight* section is on research on the alkaline activation of natural (non-waste) materials, pre-processed or otherwise (a key issue in any assessment of the future of AABs).

No-one questions the need for a complex and intense industrial process to manufacture Portland clinker: why then assume that precursors for AABs must necessarily be sourced from landfills? Why not design a universal industrial process to manufacture aluminosilicate precursors? Acknowledging that aluminosilicate precursors may be manufactured in industrial facilities is tantamount to admitting the absence of raw material limitations for manufacturing AABs worldwide.

The following idea is of particular interest in a scenario where the AAB precursor is a manufactured product rather than material collected in a landfill and used with no pre-processing: Blast furnace slag (Ben Haha et al., 2011a; Criado et al., 2018; Cheah et al., 2021), fly ash (Singh 2018; Abdullah et al., 2020), a wide range of industrial, mining and agroforestry wastes (Matalkah et al., 2016; Rivera et al., 2020; Yliniemi et al., 2020), metakaolin (Favier et al., 2014; Dupuy et al., 2019; Biel et al., 2020), dehydroxylated clays other than metakaolin (Marsh et al., 2018; García-Lodeiro et al., 2015c; Garcia-Lodeiro et al., 2018; Khalifa et al., 2020; D’Elia et al., 2020), and other natural materials such as volcanic ash, natural pozzolans and similar (Robayo-Salazar et al., 2019; Occhipinti et al., 2020) all share one valuable characteristic: a vitreous/amorphous component highly reactive with alkalis that accounts for a large fraction of their mineralogy. That vitreous/amorphous phase, containing variable proportions of Si, Al and Ca, is the key agent in the precipitation of cementitious gels such as N-A-S-H, N-(C)-A-S-H and (N)-C-A-S-H when the precursor is mixed with water and alkalis (Wu et al., 2019; Li et al., 2020). To draw a parallel with PC, precursors may be said to be equivalent to clinker, the product formed in heat-intensive cement kilns processes (Richardson and Taylor, 2018).

At this writing, there are at least two technological options for harnessing abundant mineral resources, which would make AABs universally viable construction materials:

A: industrial production of (non-molten) amorphous (*) precursors consisting in dehydroxylating clays at 500°C–800°C; B: industrial production of vitreous precursors (*) based on the total or partial fusion of blends containing clay and other minerals at 1,000°C–1,200°C (simulating materials with compositions similar to those of fly ash or slag).

Transitioning from an energy-intensive but well-known and universally accepted system such as used to produce clinker to a likewise energy-intensive but less technically tested system to produce AAB precursors might seem futile. Such a transition would be beneficial in terms of sustainability, however, for three reasons.

1) As clays and feldspars and aluminosilicate minerals in general contain barely any carbonates, their combustion entails scantly any CO_2_ emissions (in PC, 60% of the CO_2_ emitted is attributable to limestone decarbonation).
2) The thermal treatment required to dehydroxylate a clay is of low to medium intensity (500°C to 800°C). The temperatures required to (wholly or partially) vitrify a blend of clay and natural fluxes (1000°C to 1200°C) is also much lower than needed to manufacture clinker (1.400°C–1.500°C).
3) Mining clays and minerals other than limestone would contribute to geological equilibrium, presently skewed toward the extraction of calcareous deposits.
*) Although the terms ‘vitreous’ or ‘glass’ and “amorphous” are normally used indistinctly to describe non-crystalline solids, this article adopts Gupta’s premise: ‘amorphous and vitreous are two mutually exclusive states’ (Gupta, 1996).

Processing clays to manufacture aluminosilicate precursors is consequently more sustainable but not more complex or costly than manufacturing clinker by processing calcareous minerals.

Two additional considerations are in order. 1) The technology required to produce (non-molten) amorphous precursors or even (molten) vitreous precursors on the industrial scale is known and would not require significant short-term financial investment in technological innovation. In fact, most of the technology is already in place at any PC plant. 2) Developing procedures to use clays and other abundant minerals to produce AABs does not preclude the use of waste apt for that purpose.

#### Alkaline Activation of Non-vitreous Clay Precursors

As essential components in soil, clays are in abundant supply on the Earth’s crust. The variety of clay minerals on the Earth’s crust is so vast (Ito and Wagai, 2017), however, that some should reasonably be assumed to be alkali-activatable (it is a matter of statistics). Today’s cement industry acknowledges calcined clays as possible supplementary cementitious materials (SCMs) that could replace fly ash and blast furnace slag in PC manufacture (Skibsted and Snellings, 2019).

**FIGURE 3:**
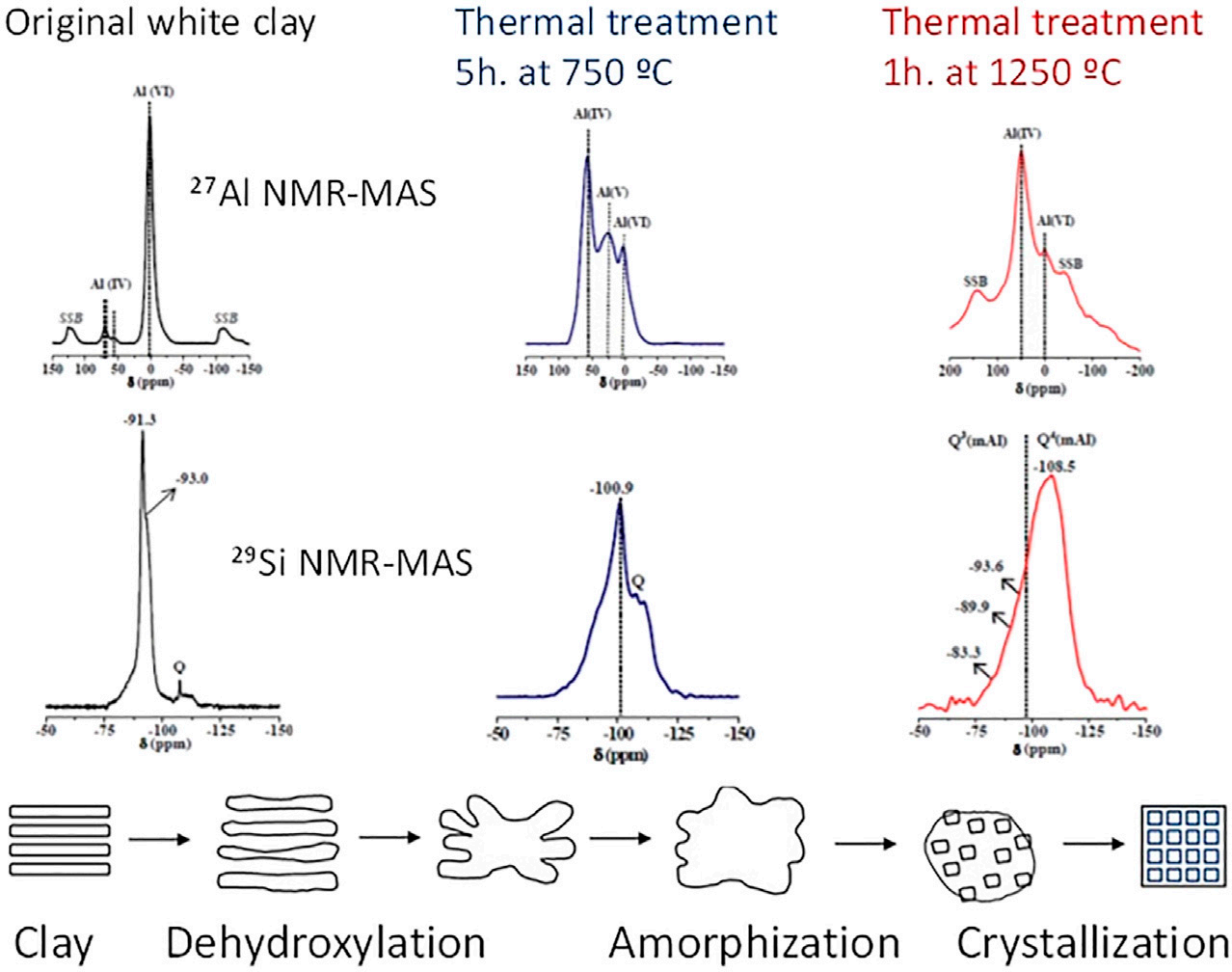
Variation in clay structure with temperature (designed by the authors) based on the NMR spectra data published by Ruiz-Santaquiteria (2013a) and Skibsted and Snellings (2019).

Clay geology conditions its chemistry and mineralogy and therefore largely its reactivity, which can be stimulated with heat (500– 800°C), to convert all or part of a clay into an amorphous material. Dehydroxylation substantially alters the spatial arrangement of clay atoms, modifying the Al coordination number (Valentini, 2018) and the degree of Si polymerisation (Madani et al., 1990). The ^27^Al and ^29^Si, NMR findings in Figure 3 illustrate the significant changes taking place in a clay nanostructure after heating at 750°C and 1250°C (Ruiz-Santaquiteria, 2013a). More intense thermal processing can induce recovery of the material’s structural order (Skibsted and Snellings, 2019).

Another option for improving clay mineral reactivity is mechanical processing. Grinding reduces particle size and may likewise contribute to the nanostructural disorder in crystalline networks (Hounsi et al., 2013), although it is unlikely to induce full amorphisation. Tole et al. (2019) recently published a useful analysis of the mechanical treatment of clays. The main purpose of grinding is to convey as much energy as possible to the material, for the greater the energy accumulating on the surface of the particles the greater is the chemical reactivity of the processed materials. That energy adopts the form of dislocations and other surface defects (Baláž et al., 2013) or even fusion bridges induced by particle interpenetration and the appearance of a thin liquid film on the surface (Juhász and Opoczky, 1990). As mechanical processes have sufficient energy to break O-H, Al-OH, Al-O-Si and Si-O bonds (Frost et al., 2001), they can prompt substantial structural alteration in crystals. For example, mechanical grinding of an undehydroxylated kaolinite-type clay was shown to produce an amorphous material fully consistent with the same dehydroxylated clay in terms of its Si and Al coordination states (MacKenzie et al. 2007), providing the grinding is sufficiently vigorous.

Although Onsager (1931) suggested that thermal and mechanical activation affect clay similarly, they differ in two ways. 1) Mechanical treatments take place under non-equilibrium conditions (Berry et al., 2004); and 2) temperature is a thermodynamic variable whereas mechanical deformation has only local effects (Cahn and Haasen, 1996). Those differences infer that mechanical and thermal activation must induce different types of behavioural change in treated clays. Comparing the efficacy of thermal and mechanical-chemical activation of a kaolin in terms of susceptibility to alkaline activation, Balczar et al. (2016) observed that mechanical-chemical activation may be a very effective method for producing geopolymers.

Clay mineral phases can also be amorphised with chemical procedures involving partial dissolution. Chemical attack may add to particle surface reactivity by generating reticular defects (Komadel, 2003); even if some author like MacKenzie et al. 2007, reported that the effect of the acid treatment on undehydroxylated kaolinite-type clay brings about little change in the XRD and NMR characteristics of the clay.

Successively combining different processes (thermal, mechanical, chemical, etc.,) is another approach tested by other several authors (Vizcayno et al., 2010).

The two most common and representative clay minerals are kaolinite [Si_2_Al_2_O_5_ (OH)_4_; type 1:1] and montmorillonite (Mx nH_2_O) [Al_2_–xMgx)Si_4_O_10_(OH)_2_; type 2:1, where M = an interlayer cation]. Many studies have ranked clay reactivity as follows: kaolinite > Ca-montmorillonite > illite > hectorite (Fernandez et al., 2011; Hollanders et al., 2016). By way of a general rule of thumb, metakaolinite is more reactive than any calcined 2:1 clay (Garg and Skibsted, 2019).

The chemistry and physics of the contact between metakaolin and alkalis (chemical reaction diversity, nature and kinetics; paste rheology; reaction product porosity and so on) are so peculiar that alkaline activation, a fairly narrow field of endeavour, has given rise to very intense scientific research, naturally attested to in detail in the literature (Lin et al., 2020; Ambikakumari Sanalkumar et al., 2021). The understanding acquired to date on the alkaline activation of metakaolin is applicable to any metakaolin anywhere in the world. Consequently, geopolymers manufactured with very pure metakaolin feature very uniform, highly predictable properties (Li et al., 2010; Duxson and Mallicoat et al., 2007a; Medri et al., 2010; Mo et al., 2014; Granizo et al., 2002, 2014). Many authors have nonetheless based their research on the use of low purity clay to manufacture AAMs (García-Lodeiro et al., 2015c; Ruiz-Santaquiteria et al., 2013b; D’Elia et al., 2020), for exactly the same reasons as authors studying LC3 cements (Akindahunsi et al., 2020; Martirena-Hernandez, 2020).

Buchwald et al. (2009), for instance, conducted several studies on smectite and smectite/illite-like clay aptness for alkaline activation. They concluded that when thermally activated such materials are partially solubilised in basic media (6M NaOH), yielding a material that hardens after moderate (60°C) thermal curing. Clay reactivity (amount of silica and alumina solubilised) in basic media and the type of end products obtained appear to be highly sensitive to the thermal pre-treatment applied.

MacKenzie et al. (2008) when studying the geopolymer formation from 2:1 aluminosilicate minerals observed that neither the undehydroxylated or dehydroxylated mineral forms a viable geopolymer unless a vigorous grinding of the original mineral takes place.

Xu and Van Deventer (2000) in turn, explored the use of 16 natural aluminosilicate minerals with different structures and compositions (illite, sillimanite, andalusite and others) as potential sources of silicon and aluminium in alkaline activation. The conclusion drawn was that they all solubilised to a lesser or greater extent in basic media (more intensely as a rule where NaOH rather than KOH was used). They were also observed to develop mechanical strength ranging from 2.5 to 19 MPa after curing for 72 h at 35°C, depending on the composition and structure of the mineral at issue, its solubility in basic media and the cation used in the activator.

Any number of generally high quality papers have described and discussed clay activation-based AAB production. An extensive review can be found in (Khalifa et al., 2020), where the conclusion drawn is that dehydroxylated clays may constitute the mainstay of an excellent procedure for preparing AABs.

#### Alkaline Activation of Synthetic Vitreous Precursors

The scientific literature identifies three types of materials in connection with the Alkaline Activation of synthetic vitreous precursors for producing strong and durable cements:

- vitreous urban/industrial waste
- laboratory reagents, with à la carte design of optimal glass composition
-  natural raw materials to prepare universal vitreous precursors     (flyglass*)

* flyglass: Term coined by authors of this article to define a glass with a composition similar to that of the vitreous fraction of a type F fly ash.

##### Alkaline Activation of Vitreous Waste

A number of types of waste with different chemical compositions, including fused silica glass, sodium borosilicate glass (over 90% of output), lead oxide glass, aluminosilicate glass and germanium oxide glass, are marketed and hence available to the scientific community for research (Shi and Zheng, 2007; Lu and Poon, 2018; Jiang et al., 2019; Wang et al., 2016). Such glass normally contains 60–95% silica (SiO_2_) and 5–10% Na_2_O, with some (0–15%) CaO and possibly <5% Al_2_O_3_. The alkaline activation of urban and industrial glass waste (bottles, windows and miscellaneous items) is described in the literature (Torres-Carrasco et al., 2017). The post-grinding activation of such glass yields compact and mechanically strong matrices (with 7 days mechanical strength of up to 56 MPa in some cases), although the scant aluminium content in these materials may compromise end product durability.

For that reason, some authors envisage the use of vitreous waste as cement additions. Zhang et al. (2017), studying the use of glass as a partial replacement for certain components in alkali-activated slag/ash systems, concluded that glass powder features high reactivity with alkalis at ambient temperature. The predominant reaction product was a C-(N)-A-S-H) gel. The alkaline activation of glass powder-bearing materials has been successfully tested in the pilot manufacture of a number of construction materials, including tiles (Rivera et al., 2018), blocks (Lu and Poon, 2018) and air-entrained concrete (Bădănoiu et al., 2015). For further details on the use of glass waste as an AAB precursor, see the review by Liu et al. (2019).

This article began by claiming that waste, no matter how suitable, abundant or inexpensive, cannot constitute the key (the only one) component in a uniform and universal industry. Urban glass, no exception to that rule, should therefore only be used as a material of unquestionable local interest able to reduce and rationalize natural raw material consumption (circular economy), but never as a raw material on which to build an industry as important as cement.

##### Pure Reagent Blends: Vitrification and Subsequent Alkaline Activation

**FIGURE 4:**
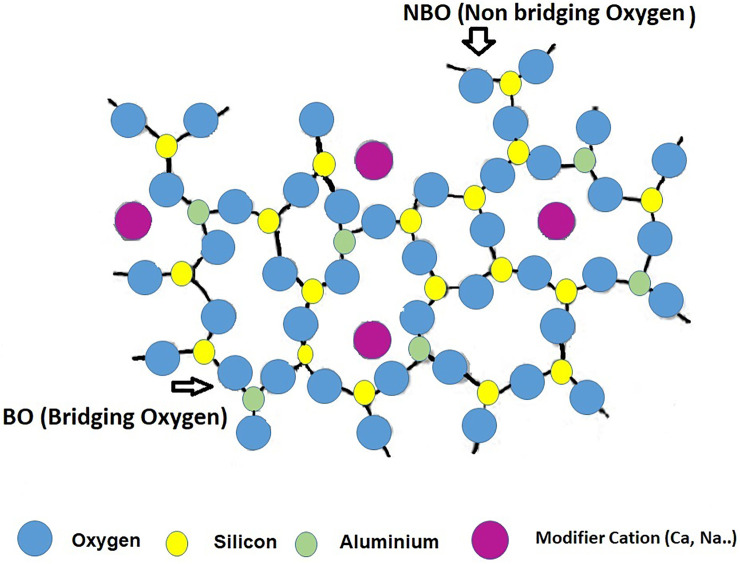
Schematic two-dimensional illustration of aluminosilicate glassy phase (Designed by the authors).

The most prominent consequence to be drawn from the preceding sub-section may be that glass formulation may be understood and programmed as a flexible exercise that addresses two challenges: 1) optimizing glass composition for subsequent use as a precursor (flyglass); and 2) minimizing fluxing temperatures. Several studies have been conducted on reactive glass manufacture for possible use as SCMs or AAB precursors (Rajaokarivony-Andriambololona et al., 1990; Garcia-Lodeiro et al., 2014, 2016a; Newlands et al., 2017; Schöler et al., 2017; Thomsen et al., 2017; Golek et al., 2019; Kucharczyk et al., 2019; Nie et al., 2020), with (variable) compositions located on the Na_2_O-SiO_2_-Al_2_O_3_ and CaO-SiO_2_-Al_2_O_3_ ternary diagrams. All aimed to develop a universal procedure for producing a uniform and optimal glass, simulating the formation of glass with compositions similar to that of fly ash and/or slag. That material would be characterised by a polymerised, tetrahedrally coordinated silica and alumina network, with both elements acting as network generators (Figure 4). Alkaline and alkaline-earth cations (Na^+^ or Ca^2+^) would act as network modifiers *via* ion bonds, the weakest part of the structure and glass reactivity would depend essentially on the degree of polymerisation. Inasmuch as the inclusion of network modifiers favours depolymerisation and therefore the formation of non-bridging oxygen atoms (NBO), it has a beneficial effect on reactivity.

**FIGURE 5:**
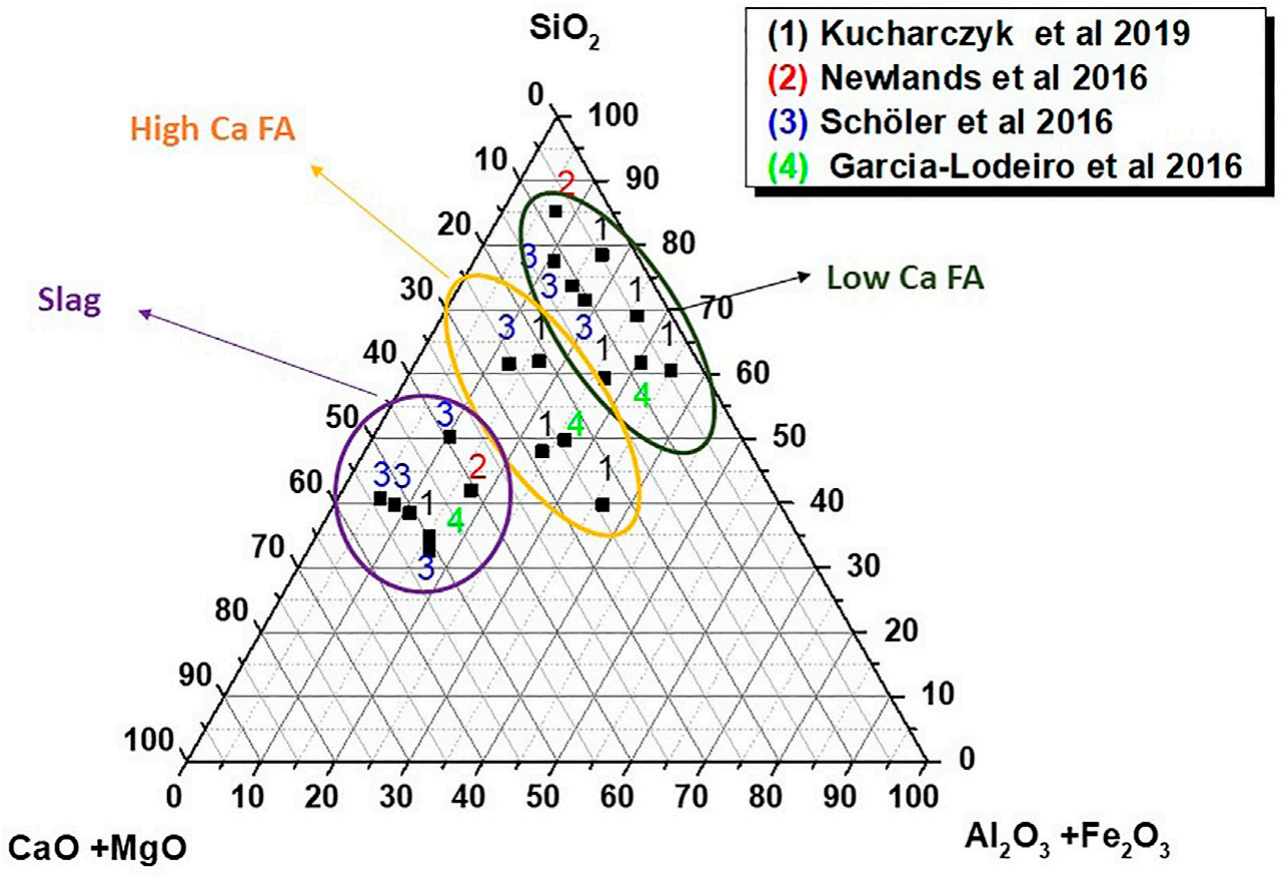
Chemical composition of synthetic glass studied by several authors (designed by the authors) based on information drawn from articles by Kucharczyk et al., 2019; Newlands et al., 2017; Scholer et al., 2017; Garcia-Lodeiro et al., 2016a).

Figure 5 depicts the compositions of synthetic glass formulated by thermally treating stoichiometric mixes of laboratory reagents CaO, Al_2_O_3_, SiO_2_, and NaOH. Whilst some authors have associated the most reactive glass compositions with the highest CaO contents (Garcia-Lodeiro et al., 2016a; Newlands et al., 2017), the presence of Al_2_O_3_ in the glass structure has also been observed to be essential to reactivity (Schöler et al., 2017; Kucharczyk et al., 2019).

**FIGURE 6:**
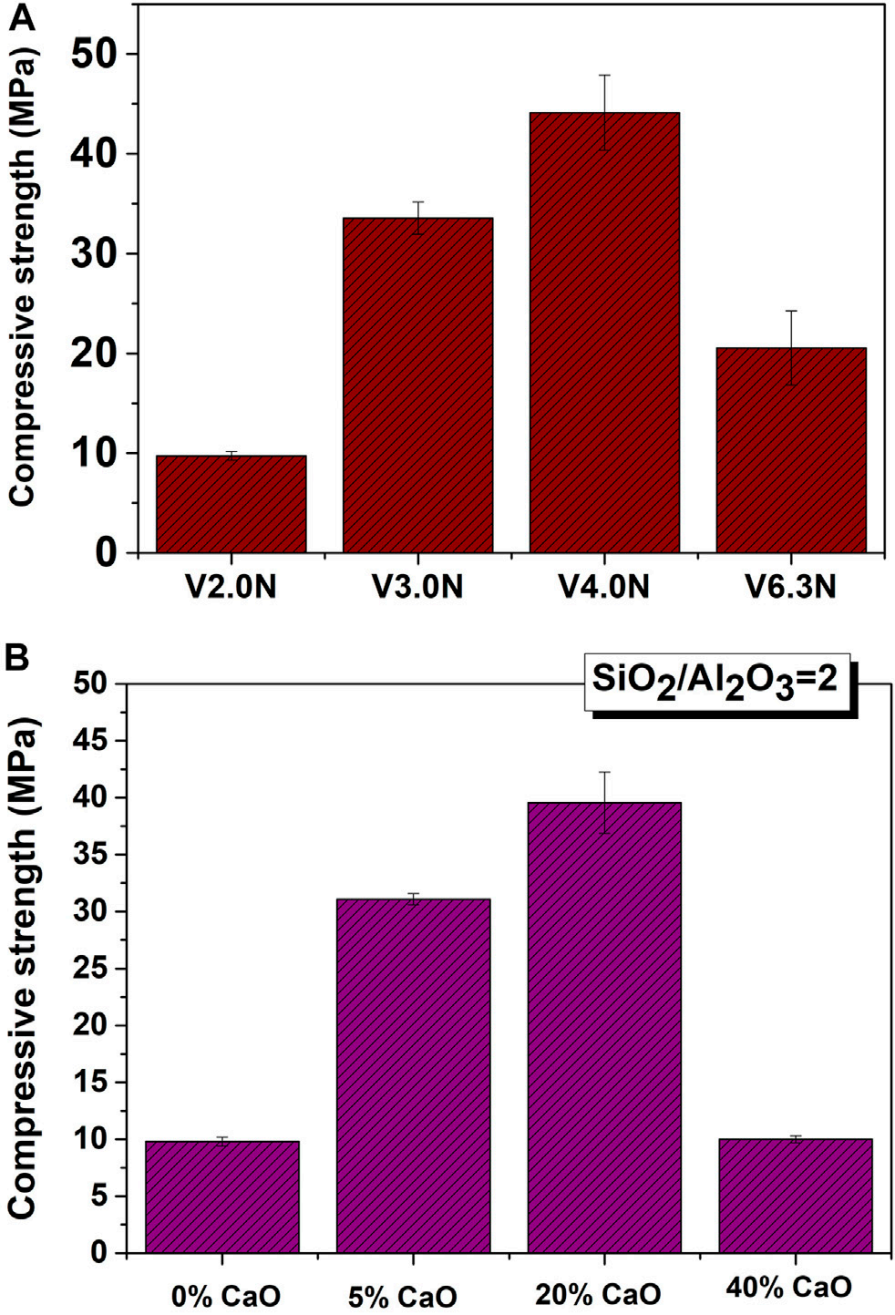
Compressive Strength development in alkali-activated synthetic glass (20 h, 85°C, 8M NaOH) with: **(A)** different SiO_2_/Al_2_O_3_ ratios; **(B)** the same ratio and different CaO contents (*Legend:* N represents glasses activated with NaOH, and V is the nomenclature for synthetic glass prepared with different S/A ratios).

Garcia-Lodeiro et al. (2014), Garcia-Lodeiro et al. (2016a) used an 8M NaOH solution to alkali-activate glass with different compositions (SiO_2_/Al_2_O_3_ = 2, 3, 4, and 6.3), subsequently cured at 85°C and RH>95% for 20 h. Their findings showed that glass with SiO_2_/Al_2_O_3_ ratios of 3–4 yielded pastes with strength of over 30–40 MPa (Figure 6A). The same authors (Garcia-Lodeiro et al., 2014) analysed the depolymerising effect of calcium on glass structure and its implications for reactivity. Glass synthesized with a SiO_2_/Al_2_O_3_ ratio of ∼2 and variable (0%, 5%, 20% or 40%) CaO content was activated under the above conditions (8M NaOH, 20 h at 85°C and RH>95%). Reactivity rose with calcium concentration (Figure 6B) up to a threshold 20%, after which compressive strength declined substantially. Other authors synthesized glass similar to blast furnace slag (Golek et al., 2019), obtaining compressive strength values of up to 100 MPa. In glass as in other precursors, whilst chemical composition is important for optimal strength development, activating conditions (type and concentration of activator, temperature, time, humidity) are likewise important.

Alkali-activating glass yields the same reaction products as in traditional AABs. Glass with composition similar to type F fly ash generates a dense product identified as an N-A-S-H gel and zeolites (Schöler et al., 2017). In glass with a high CaO content (slag composition), the primary cementitious product generated is an (N,C)-A-S-H gel, similar to that observed in the alkaline activation of hybrid cements (Golek et al., 2019).

Synthetic aluminosilicate glass has proven to be an apt precursor, generating high-performing matrices. The composition of the starting glass and its degree of polymerisation, along with the melting temperature and suitable cooling must be carefully controlled to ensure the development of optimal precursors.

##### Alkaline Activation of Glass Prepared From Molten Blends of Natural Minerals

Studies conducted by Ruiz-Santaquiteria (2013a) constitute an excellent example of aluminosilicate glass synthesis from blends of several minerals: common clays and feldspars for the silica and alumina needed plus a small amount of limestone, used both as a flux and to modify the glass structure. The authors first studied the effect of synthesis temperature, type of flux and starting mix composition on the properties of the end product. The research by Ruiz-Santaquiteria et al. (2013b), Ruiz-Santaquiteria et al. (2016) confirmed that adding a small amount of CaO to the starting mix favoured the formation of a homogeneous material; in other words, the use of the flux may have lowered viscosity and with it the surface tension of the molten mass, preventing the latter from segregating into differentiable phases during solidification (Timashev, 1980).

The alkaline activation of the glass so synthesized from clay (Table 1) yielded strong, compact matrices (Figure 6).

**TABLE 1:** Flyglass synthesis and activation. (Ruiz-Santaquiteria, 2013a)

Name	Glass synthesis: conditions		^b^Alkaline activation (NaOH 8M)	
	^a^Starting mix composition		T^a^ (^o^C)	Compressive strength (MPa)
**A**	46.9 %BC+39.3 %KF+13.8 % Fluxes		1250	16 ± 1.9
**B**	46.9 %BC+39.3 %KF+13.8% fluxes		1400	15 ± 2.2
**C**	42.2 %BC+35.4% KF+12.4 %fluxes +10.0 %CaCO_3_		1250	64±3.1

a Ball clay (BC) and potassium feldspar (KF)

b Cured for 20 h at 85°C and RH > 95 %)

By adding just 5.6 wt% of CaO to the starting mix, glass was produced at technologically promising temperatures (1,250°C), developing matrices with compressive strengths of 65 MPa when alkali-activated. Compare that to the mean CaO content in clinker, around 65 wt%.

**FIGURE 7:**
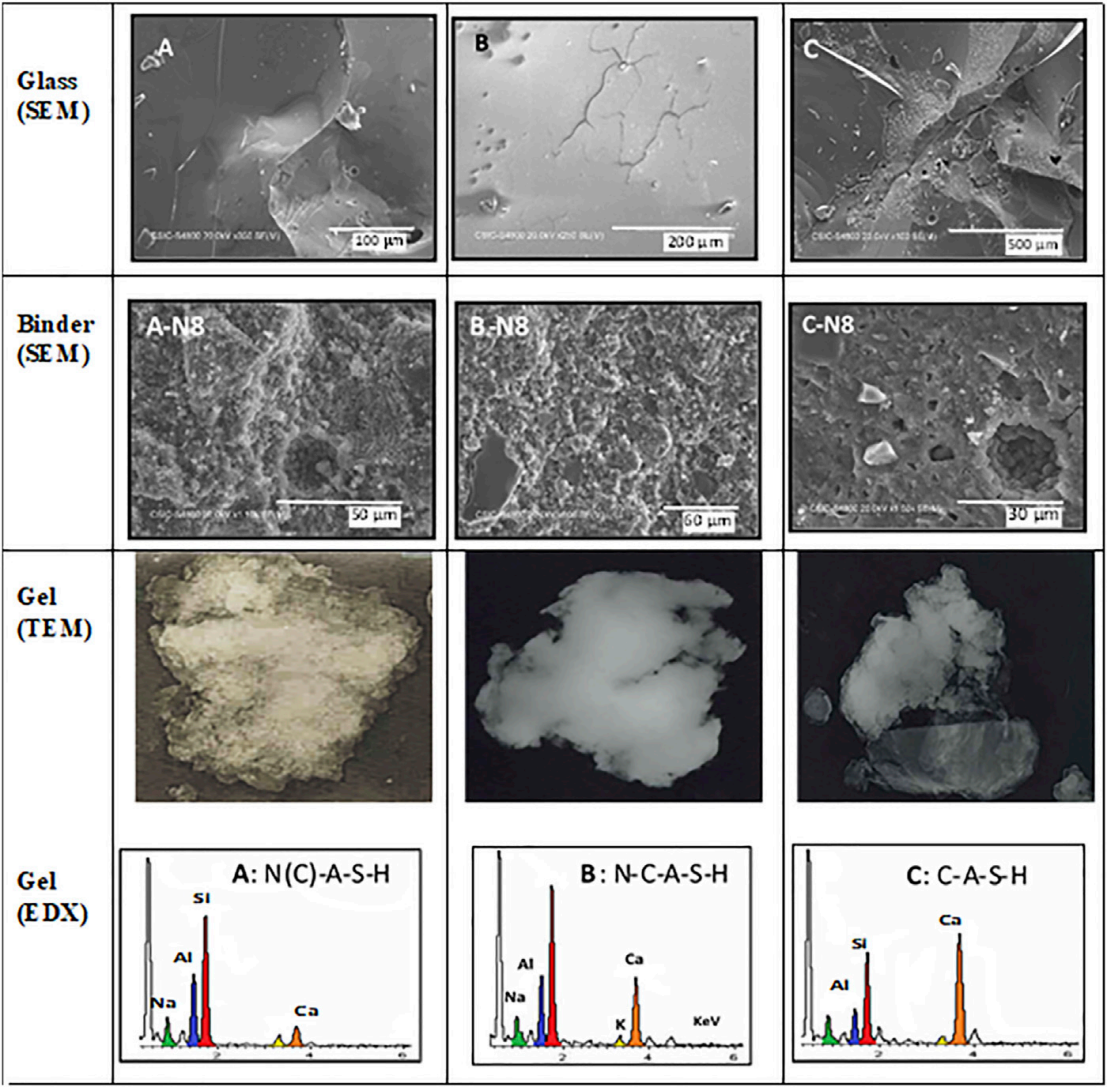
TEM micrographs and microanalyses of original and alkali-activated synthetic flyglass [adapted from Ruiz-Santaquiteria (2013a)].

Post-activation identification of the reaction products revealed that the presence of small amounts of CaO in the glass induced the precipitation of an N-C-A-S-H gel that was much more compact and mature than the N-A-S-H gel formed in the absence of calcium. The TEM micrographs of the starting glass, post-activation paste and gels and respective microanalyses reproduced in Figure 7 attest to the post-activation formation of an N-(C)-A-S-H gel in glasses A and B and C-(N)-A-S-H gel in glass C.

Some other authors (apart from those signing this article) have observed the co-existence of C-S-H and N-A-S-H gels in hybrid cements (Yang et al., 2012; Rojas-Duque et al., 2020). The prevalence of one or the other depends essentially on calcium content and system alkalinity. In the Ruiz-Santaquiteria et al. (2013b, 2016) findings, both the high alkalinity and low calcium content favoured the formation of N-A-S-H and N-(C)-A-S-H gels. Research suggests that the combination of those gels normally improves strength development in cements (Askarian et al., 2018; Fernández-Jiménez et al., 2019a).

Other authors have thermally processed and vitrified high MgO clays such as phlogopite (KMg_3_AlSi_3_O_10_ (OH) (Sreenivasan et al., 2017); moreover MacKenzie et al. 2013 had previously reported that a geopolymer was formed from a sepiolite mineral previously ground and dehydroxilated.

Magnesium, an abundant element with higher electronegative potential even than calcium, has been studied in connection with the durability of cementitious materials, including alkaline cements. Various authors, researching the effect of MgO on alkali-activated slag strength and durability (Ben Haha et al., 2011b; Shen et al., 2011), have concluded that raising the content of that oxide in cementitious blends enhances strength performance. In light of the foregoing, the feasibility of using non-carbonated sources of MgO such as these clays as an additional component in vitreous precursor preparation to ultimately generate good alkaline cements would appear to merit exploration.

In short, clay (generally speaking, the large family of aluminosilicate minerals) is an ideal source of raw materials for alkaline cement precursor design. The need for processing (amorphisation or even vitrification) is no obstacle, providing it is technologically feasible and economically cost-effective. What makes these geological resources so appealing is that their processing entails practically no CO_2_ emissions.

### Activators. Requirements to Generate High pH Conditions

The same studies that deem waste to be the archetypal aluminosilicate precursor often persist in using caustic soda and/or waterglass as the alkaline activators par excellence and consequently repeating the platitudes questioned in this paper (Van Jaarsveld and Van Deventer, 1999; Shi et al., 2015; Zuo et al., 2019; Singh and Middendorf, 2020; Perumal et al., 2021; Zhang et al., 2021). Not only are both high carbon synthetic products, but they entail some risk of injury to handlers and high costs (only economically assumable in construction under certain specific conditions).

**FIGURE 8:**
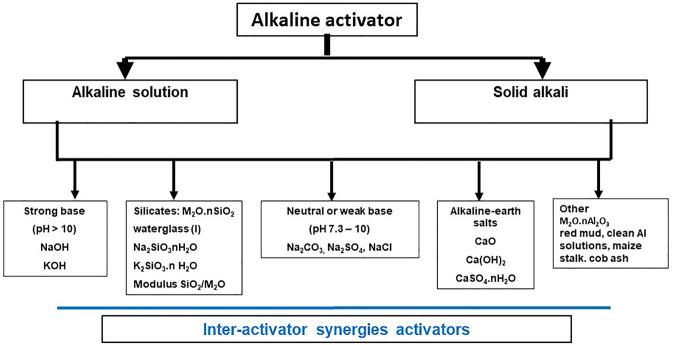
Classification of most prominent activators used in AAB preparation (designed by the authors).

Alkaline activators are as essential as precursors to AAB production. The literature identifies any number of products (Figure 8) able to catalyze the conversion of aluminosilicate precursors into strong, compact matrices. Some of the products depicted in Figure 8 are analyzed in item *Chemical Products of Prominent Use in Alkaline Activation Section* below. But before assessing the greater or lesser (technical and industrial) promise of each product, some thought is due to the role of activators in the mechanisms that govern the chemical activation reactions. Indeed, the choice of the activator best suited to each situation must always be made in keeping with the chemistry of the respective cementitious system, the expected environmental impact, the technology available to convert raw materials into precursors and the related logistic and economic considerations.

Two elements of the chemistry involved make the precursor-activator an indivisible binomial: 1) pH and 2) the roles played by anion and cation.

Solution pH conditions precursor solubility. Blast furnace slag is highly soluble in acid media, for instance, but the hydrates formed are unstable and fail to generate compact matrices (Živica and Krizma, 2013; Breitenbücher et al., 2018). Alkaline pH, in contrast, not only raises precursor solubility but favours the formation of stable hydrates that heighten material mechanical strength. A number of authors (Puertas 1993; Alonso and Palomo, 2001) have reported rising pH and hence greater system alkalinity to enhance silica and aluminium solubility in different types of precursors (Benavent et al., 2016). Depending on precursor calcium content, however, a rise in OH- ion concentration has also been observed not only to fail to alter the amount of material solubilized but even to have adverse effects. The reason is that whereas rising pH raises silica and alumina solubility, alkaline pH lowers calcium solubility. Recapitulating, the pH of solutions used to activate precursors with a high calcium content such as slag and type C fly ash must therefore be considerably lower than needed to activate low calcium aluminosilicates such as type F fly ash and metakaolin.

The activator cation plays a dual role in precursor activation, maintaining the pH of the aqueous phase at the desired level and adhering to the reaction products. Cations should be readily taken up into the structure of the main reaction product to offset the electric charge imbalance arising when a SiO_4_ tetrahedron is replaced with an AlO_4_ tetrahedron, or into other secondary reaction products such as zeolites (Belviso et al., 2017). Na or K hydroxides or salts are normally used. K compounds exhibit greater alkalinity, associated with higher precursor solubilization potential. Empirical evidence shows, however, that sodium compounds are better able to release scantly polymerized alumina and silica (Macphee and Garcia-Lodeiro, 2011; Fernández-Jiménez et al., 2013). That may be attributed to the smaller size of Na + than K+ or a higher charge density that enables the ion to travel more effectively through the precipitant gel. In high calcium precursors, less Mg is dissolved with KOH than with NaOH (Roy et al., 1992).

Ca(OH)_2_ is the alkaline-earth salt activator most frequently used. The resulting solutions generate a pH∼12.5, which would explain slow precursor dissolution. The presence of Ca^2+^ in the system has other significant implications, however, for it may be taken up into the cementitious gel structure. The reaction between aluminosilicates and Ca(OH)_2_ solutions is also known as the pozzolanic reaction (Richardson and Taylor, 2018).

The activator anion, in turn, may also have a sizeable effect on the reactions generating cementitious systems and therefore on the mineral and nanostructural characteristics of the reaction products. The anions normally added to the medium to activate precursors include hydroxyl groups, silicates, carbonates and, to a lesser (but not less promising) extent sulfates, nitrates, fluorides and chlorides. Those anions may be taken up into the cementitious gel or contribute to the formation of secondary products of technological interest (Fernández-Jiménez, 2000; Fernández-Jiménez, 2003; Shi et al., 2003; Xu et al., 2008).

Activators have specific technological implications insofar as they may be used in liquid or solid form, a fact of significant environmental and economic consequence. Most studies, conducted with liquid hydroxide or alkaline salt activators (Figure 8) mixed with a solid precursor, pursue aims relating to OH- concentration, type of alkaline cation or the SiO_2_/Na_2_O ratio (Fernández-Jiménez and Palomo, 2005a). Although the type most commonly used in laboratories, liquid activators may pose industrial-scale problems, as they are viscous, corrosive, hazardous, expensive and scantly sustainable and therefore applicable only to very specific construction scenarios. Many authors nonetheless unfortunately believe that alkaline activation is only effective if mediated by waterglass or caustic solutions or a mixture of the two. If that were the case, the economic and environmental viability of AABs would of course be highly questionable and this article would not be compatible with the motivation that spawned it. Some studies have identified the liquid activator dose as the critical element in determining AAB profitability (Miller et al., 2018) and actual environmental footprint (Habert et al., 2011).

Solid activators are an option, however (Nematollahi et al., 2017), for one or several precursors can be mixed or jointly ground with one or several solid activators. The procedure, similar to PC production and hydration processes, consists in preparing a dry blend and subsequently adding mixing water. Calorimetric studies have shown that in such cements activator dissolution is nearly instantaneous and followed by the reactions involved in aluminosilicate dissolution and concomitant reaction product precipitation (Fernández-Jiménez et al., 2019a). Some authors (Abdullah et al., 2012; Shi et al., 2019) have reported that solid alkaline salts, which are very abundant in seawater and on the Earth’s crust, can mediate in generating the pH required to alkali-activate aluminosilicate precursors.

The following is a brief summary of some considerations around the alkaline activators most commonly used at present and past, together with those that should desirably be prioritized in the near future.

#### Chemical Products of Prominent Use in Alkaline Activation

As Figure 8 shows, a wide variety of products, many amply described in the literature, can be used as alkaline activators. This item discusses the ones deemed of greatest scientific and industrial interest.

CO_2_ emissions associated with the production of commonly used alkaline activators (NaOH and waterglass mainly) were determined by S.A. Miller et al. 2018. Because different raw material sources, and processing can be implemented in the production of those activators, a range of emissions was considered by the mentioned author. This comment is an important one, taking into account that it is the base of a negative environmental characteristic of many AABs. The positive fact is that there exist some abundant natural products (alkaline salts like Na_2_CO_3_, Na_2_SO_4_, NaCl, etc) with almost no carbon footprint which have demonstrated to be useful and effective in the Alkaline Activation processes.

##### Strong Bases (pH > 14): NaOH, KOH

Hosts of studies have been published on the use of the caustic hydroxides NaOH and KOH as alkaline activators. Their industrial use is only exceptionally practical however, for economic, safety and environmental reasons. Technically speaking, 8–12 M caustic solutions are recommended for low calcium precursors (Palomo et al., 2014) and lower concentrations for precursors with medium-high calcium content. Concentrations of 3–5 M have been recommended to activate slag (Fernández-Jiménez, 2000), whilst some authors have applied solid-state NaOH flakes (Suwan and Fan, 2017). In the latter case, the precursor is mixed with soda at ambient temperature and water-hydrated, after which the mix sets and hardens, although the strength attained is normally lower than when dissolved soda is used. Sodium hydroxide is ideal for use in research (where it is the benchmark activator) because while ensuring a broad range of pH it can also be used to assess the quality of individual precursors without generating secondary chemical reactions often difficult to study.

##### Silicates: M_2_OnSiO_2_

Potassium or sodium silicate has been used as activators in a number of studies. Although sodium silicate is deemed by many authors as the key activator for preparing AABs, as it induces the formation of very high mechanical strength matrices, its use may pose paste workability (Palomo et al., 2005), rapid setting and drying shrinkage problems (Wang et al., 1995).

Sodium silicate owes its large carbon footprint to how it is generally synthesised: fusing siliceous sand with anhydrous sodium carbonate (Na_2_CO_3_) at temperatures of >1.000°C (although also by directly attacking silica with caustic soda) (Lagaly et al., 2000). The result is a hard material that can be commercialized as a granular solid or an aqueous liquid.

A significant share of the studies published on alkaline activation are based on the use of liquid sodium silicate. Soluble silicates have a dual effect on AA, contributing to: 1) necessary system alkalinity; and 2) the formation of a high silica gel. Alkali concentration as well as the SiO_2_/Na_2_O ratio can be readily modified either by diluting the solution with water or adding extra alkalis to adjust the pH and silica polymerization and with it activator efficacy. Promising studies on the use of solid sodium silicate have also been described in the literature (Wang et al., 2017).

##### Neutral or Weak Bases (pH 7.3—10): Na_2_CO_3,_ Na_2_SO_4_, and NaCl

The use of carbon footprint-free natural Na_2_CO_3_ (natron) to manufacture AAB may be deemed environmentally sustainable. That most of the Na_2_CO_3_ used is synthetic, however, belies such carbon neutrality to some extent. Even so, the environmental impact of sodium carbonate is substantially lower than that of caustic solutions or waterglass. Its price may vary from country to country, but may be regarded as affordable if dosed within certain limits.

Na_2_CO_3_ induces a lower pH (∼11.5) than the activators described above, which is potentially beneficial in terms of health and safety concerns. Given that a low pH may retard initial hardening and strength development in AABs, however, Na_2_CO_3_ may have received less attention than it merits. A fair number of examples of its use both in solution and a solid can be found in the literature (Shi et al., 2003; Fernández-Jiménez and Palomo, 2019a; Provis et al., 2014). As a solid it has been successfully applied in hybrid alkaline cements (Peng et al., 2017). The use of K_2_CO_3_, in turn, has similar effects and is less susceptible to efflorescence formation (Askarian et al., 2018). It works well in hybrid cements, whilst the rapid setting possibly induced can be controlled with citric acid.

Sodium sulfate is an abundant natural substance (Kostick, 1993), although Na_2_SO_4_ also exists in synthetic form as an industrial by-product (McIlveen and Cheek, 1994). It is more expensive than standard cement industry raw materials but less than sodium carbonate and silicate. If its presence in binder design does not exceed certain limits and if it can be made to help the precursor develop good technological properties while contributing to AAB sustainability, its profitability is ensured. Several research groups (Donatello et al., 2013; Garcia-Lodeiro et al., 2015a; Qu et al., 2016) have analyzed the effect of Na_2_SO_4_ on the alkaline activation of fly ash and other precursors. In that context it has been applied for some time to improve lime-pozzolan cement (LPC) reactivity (Shi and Day, 1993). More recently it has acquired importance in preparing so-called hybrid cements, low-clinker binders with 70–80% aluminosilicates (Garcia-Lodeiro et al., 2013a).

Some authors have studied the use of NaCl or seawater as activators (Palomo et al., 2019), in light of salt reactivity with calcium hydroxide to form NaOH in situ (the respective reactions are discussed in greater detail in later items). Generally speaking, common salt favors both early- and late-age mechanical strength in cements with high aluminosilicate contents. Cl-ions also stabilize ettringite formation (Kishar et al., 2013; Shi et al., 2017), thereby improving early-age strength in alkaline cements. The recommended dose is 1–4% but not higher to elude the risk of concrete reinforcement corrosion.

Other neutral sodium and/or potassium salts besides Na_2_CO_3_, Na_2_SO_4_ or NaCl are or could be promising, primarily for their possible interaction with calcium salts to generate high *in situ* alkalinity (Askarian et al., 2018).

##### Alkaline Earth Products: CaO, Ca(OH)_2_, CaSO_4_.nH_2_O

Quicklime (CaO) and hydrated lime [Ca(OH)_2_] are products long known and used in construction as binders. Ca(OH)_2_ is used in alkaline cements for reasons that differ from those given for the strong/weak bases and silicates. Hydrated lime can be mixed with aluminosilicate precursors at up to 10–15% with no adverse environmental impact and can (and should) be used together with other activators to jointly generate high pH. In 2001 Alonso and Palomo (2001) studied the effect of raising the alkalinity in blends containing 50% metakaolin +50% Ca(OH)_2_. At NaOH concentrations of 5 M or lower, the degree of reaction in MK was low, with C-S-H gel forming as the primary reaction product. At higher molarity (10 M), however, MK dissolved rapidly and the prevalent reaction product was a N-A-S-H gel. More recently other authors have stressed the importance of the correlation between CaO and pH in C-A-S-H/N-A-S-H and C,N-A-S-H/C-(N)-A-S-H gel precipitation (García-Lodeiro et al., 2011). In that vein, any number of studies have been published on mixes bearing 10% metakaolin and 20% Ca(OH)_2_ (Guo and Shi, 2015).

Calcium sulfate, in turn, may appear as gypsum, basanite or anhydrite. For the purposes of alkaline activation, gypsum does not induce high pH media (pH∼8–9), but may be a promising source of calcium and sulfate ions. It yields results similar to Ca (OH)_2_ in slag (Fernández-Jiménez et al., 1996) and ash (Ma and Brown, 1997) activation.

The literature (Fernández-Jiménez et al., 2017 *;* Garcia Lodeiro et al., 2020) describes many other alternatives (such as red mud, clean Al solutions, maize stalk, cob ash, etc.), which while delivering promising (mostly laboratory-scale) results are more seldom used and hence not documented in this paper.

#### Synergies Between Neutral or Weak Bases and Alkaline Earth Salts

Water-dissolved salts undergo hydrolysis, a well-understood process involving dissociation into their respective anions and cations. Hydrolysis of a neutral salt formed from a strong acid or base may alter medium pH as a result of synergies among the reactions taking place during hydration. Justnes and Østnor (2014) proposed a general equation to describe the process:

$${\mathrm{xCa}\left(\mathrm{OH}\right)}_{2}\;+\text{ 2}\left(\mathrm{Na,K}\right)\mathrm{xA}\to {\mathrm{CaxA}}_{2}\left(s\right)\;+\text{ 2x}\left(\mathrm{Na,K}\right)\mathrm{OH}$$ (1)

Some of the salts mentioned earlier (Na_2_CO_3_, Na_2_SO_4_) can be used as a source of alkalis. Ca(OH)_2_ may be sourced externally or internally, as in hybrid alkaline cements (Palomo et al., 2019). Greater or lesser efficacy depends in part on the solubility of the calcium salt that precipitates. This process can be induced by adding dissolved activators to the mixing water or as solids ground jointly with the precursor. The reactions deemed most relevant to the present discussion are described below.

##### A) Calcium + Sulfates

Shi and Day (1993) showed that adding Na_2_SO_4_ to systems bearing 20% Ca(OH)_2_ and 80% ash enhanced early age strength substantially. Lee et al. (2003), in turn, observed that adding Na_2_SO_4_ to blends with 40% fly ash and 60% OPC hastened 1 day strength development, whereas the 28 days values were similar irrespective of the presence of the activator. Donatello et al. (2013) observed that Na_2_SO_4_ in blends with 80% FA + 20% OPC accelerated fly ash reactivity with no adverse effect on Portland clinker hydration.

In 2014 Justnes and Østnor (2014) tested variations in pH induced by Na_2_SO_4_ in a Portlandite-bearing medium. Mixing 0.2 mol of CaO with 200 ml of water yielded a solution pH of 12.55 which the addition of Na_2_SO_4_ raised to 13.2, verifying Eq. (2)

$${\mathrm{Na}}_{2}{\mathrm{SO}}_{4}{\;+\mathrm{Ca}\left(\mathrm{OH}\right)}_{2}{+\;2H}_{2}O\to {\mathrm{CaSO}}_{4}{\text{.}2H}_{2}O+\;2\mathrm{NaOH}$$ (2)

Nonetheless, gypsum is not generally detected in hybrid alkaline cements, for the medium also contains aluminate ions that react with (SO_4_)^2−^ to form AFm or AFt [Eq. 3]

$${\mathrm{xCa}(\mathrm{OH})}_{2}{\;+n[\mathrm{SO}}_{4}{)}^{2-}{]\;+m[\mathrm{Al}(\mathrm{OH})}_{4^{-}}{]\;+\mathrm{zH}}_{2}O\to \;3\text{CaO}{\text{.Al}}_{2}{\text{O}}_{3}{\text{.}3\text{CaSO}}_{4}{\text{.}32\text{H}}_{2}\text{O}$$ (3)

U-phase [Eq. (4)] has been identified when studying hybrid alkaline cements with Na_2_SO_4_ as the activator (Arbi et al., 2013; Garcia-Lodeiro et al., 2016b; Fernández-Jiménez et al., 2019a). The presence of metastable U-phase is an indirect indication of high system alkalinity (pH>13).

$${\mathrm{xCa}(\mathrm{OH})}_{2}{\;+\mathrm{nNa}}_{2}{\mathrm{SO}}_{4}{\;+m[\mathrm{Al}(\mathrm{OH})}_{4^{-}}{]\;+\mathrm{zH}}_{2}O\to \;4\mathrm{CaO}\text{. 0}{\text{.}9\mathrm{Al}}_{2}O_{3}\text{. }1{\text{.}1\mathrm{SO}}_{3}\text{. 0}{\text{.}5\mathrm{Na}}_{2}O{\text{. }16H}_{2}O(U-\mathrm{phase})\;\to {\mathrm{CaO}}_{3}{\mathrm{Al}}_{2}O_{3}{\;3\mathrm{CaSO}}_{4}{\text{. }32H}_{2}O(\mathrm{AFt})$$ (4)

Fernández-Jiménez et al. (2019a) recently studied the efficacy of fully dissolving the chemical activator in the mixing water or grinding it jointly with fly ash. Their findings showed that cement strength developed well with both procedures but better with the solid-state activator. The liquid activator shortened setting times, however, and lowered heat of hydration. Chemical activator format consequently affects reaction kinetics from the outset and ultimately the nature and composition of the reaction products formed.

##### B) Na_2_CO_3_ + Ca(OH)_2_

In these systems Portlandite (pH∼12.5) reacts with sodium carbonate to form calcium carbonate and soda as per Eq. (5), generating a pH>13, turn hastening the rate of dissolution and reaction of the aluminosilicate precursor used.

Na2CO3 +Ca(OH)2 → CaCO3 +2 NaOH (5)

Garcia-Lodeiro et al. (2013b) studied hydration in 70% FA + 30% OPC blends with 2M Na_2_CO_3_, observing gaylussite (Na_2_Ca(CO_3_)_2_.5H_2_O), along with calcite, formation in the early stages. Gaylussite, a metastable phase that temporarily blocks the effect of alkalis, slightly retards alkaline activation in aluminosilicate precursors. Its subsequent dissolution in the medium raises sodium, calcium and carbonate ion content, along with pH, favouring ash activation reactions. In 2015 Garcia-Lodeiro et al. (2015b), comparing the effect of Na_2_CO_3_ and Na_2_SO_4_, observed the former to deliver higher mechanical strength.

In short, the alkaline solutions most widely used in AAMs (caustic soda and/or waterglass) are not strictly necessary to activate precursors. More than that, in light of the synergies shown in the *Synergies Between Neutral or Weak Bases and Alkaline Earth Salts* Section, the use of neutral salts is a very practical and interesting alternative for the alkaline activation of certain types of calcium aluminosilicate precursors. Solid alkaline salts, in turn, sidestep the technological limitations associated with handling corrosive solutions, lower costs substantially and above all practically eliminate the environmental impact of synthetic products.

## Raw Material Processing. Industrial-Scale Alkali Activated Binders Manufacture

One of the most promising characteristics of AABs is the versatility of the processes involved in their production, an indisputable industrial advantage over Portland cement-based products, limited to a single, clinker-based production stream. Whilst the literature focuses primarily on the description of alkaline concrete production from copious waste (fly ash and blast furnace slag) and liquid activators, it also addresses other options that promise at least the same practical, environmental and economic benefits as the traditional alternatives. The most prominent options set out in the literature are analyzed below.

### Two-Part Binders (With Liquid Alkaline Activator)

**FIGURE 9:**
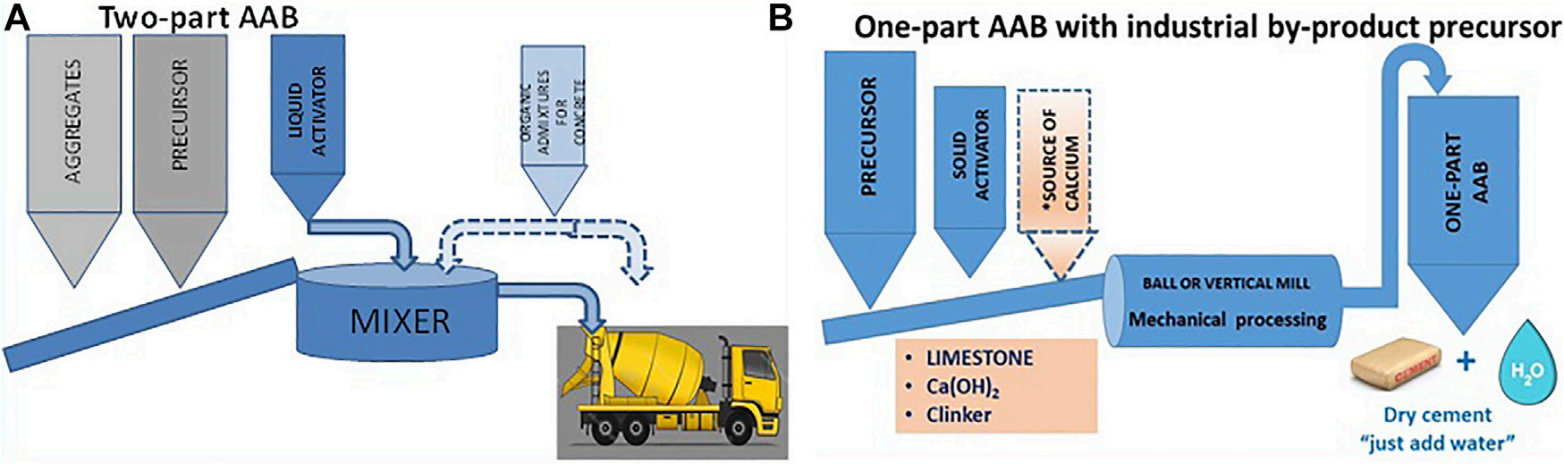
AAB flow charts: **(A)** two-part formulation; **(B)** industrial by-product-based one-part formulation.

This option (Figure 9A) is characterized by the need for two basic components to generate a compact matrix: a solid precursor and a liquid activator, there being no parallel or analogous technology for PC. With this procedure, concrete can be manufactured simply and directly with waste of different origins (both precursor and activator may be 100% by-products from other industries), enabling builders to implement construction projects with no need to acquire PC.

This approach may be deemed both economically and environmentally promising. As in PC concretes, all the materials involved to prepare AABs should be locally available to favour the circular economy (for shipping adds to process costs and has an adverse effect on the CO_2_ embodied in the end product). Moreover, the industrial infrastructure required to manufacture this type of AAB consists in no more than a mixer to combine the products needed to prepare the concrete. In other words, the low-moderate investment needed to industrialize and commercialize such concretes is affordable for many small and mid-sized enterprises.

Two-part binders are the option most thoroughly analyzed in the scientific literature on alkaline activation (Hwang and KuoWang, 2011; Nematollahi et al., 2015; Sassoni et al., 2016; Fang et al., 2018; Tigue et al., 2018; Fernández-Jimenez et al., 2019b; Wang et al., 2019; Fang and Zhang, 2020) and have been successfully used in a number of technological applications (Palomo et al., 2007; DB Group, 2018; ZEOBOND, 2020). One large scale example can be found in the over 30,000 m^3^ of a zero Portland cement concrete (denominated earth-friendly concrete, EFC) laid by the Australian firm Wagners for works at Brisbane West Wellcamp Airport in 2014 (ENGINEERS Australia, 2020). The concrete was used to pave 51,000 m^2^ of aircraft turning areas and to build the terminal building foundations and other civil works (WAGNERS, 2020). The precursor consisted in a blend of fly ash and blast furnace slag, although Wagners provides no information on the activator used. In 2016 Wellcamp Airport was judged the best engineering project in the Concrete Institute of Australia QLD State Branch’s Awards for Excellence and “Highly Commended” in the sustainability category in the national finals of that awards series (ACI, 2016).

The concrete used to repair Wodford West Viaduct on London’s M25 at Essex affords a similar example. According to an article published by Global Cement on January 30, 2020, DB Group and Axtell concluded the repairs in a matter of hours (M25 is the most heavily travelled road in the United Kingdom). The concrete used carried a slag precursor and an unspecified liquid activator at a ratio of 95:5. The AAB released 114 kg of CO_2_/t of concrete, which according to the builders was 77% lower than attributed to conventional PC concrete.

Likewise, worthy of mention is a recent CEMEX development commercialized as “Vertua concrete” (Cemex, 2020). The website defines Vertua ultra zero as a clinker-free geopolymer concrete featuring up to 70% less CO_2_ than standard PC-bearing CEM I concrete. CEMEX also claims that this geopolymer concrete can be used in hosts of applications and that the environmental principles underlying the Vertua low carbon range are firstly to maximize CO_2_ abatement and secondly to offset any residual CO_2_.

The major problem associated with that approach is that it fails to ensure universally uniform concrete (unlike the product based on Portland cement), given the compositional and geographic diversity of the waste generally used.

### One-Part Binders (With Solid Activator)

In the second option available with the current AAB manufacturing nous and technology, both precursor and activator are solid-state materials (Figure 9B). The binder, in other words, is a uniform powder containing all the components needed for alkaline activation to begin immediately when water is added to the AAB. After water hydration, one-part binders set and harden, like PC. The primary aim that should be sought in the manufacture of this type of binders is to ensure optimal control of end product quality, such as in the in PC production. One-part binders will become successful if the same worksite procedures can be applied as with PC: blending with aggregate and admixtures; mixing the materials to obtain a fluid homogeneous paste; casting in formwork, etc.,

Luukkonen et al. (2018) *,* in a review of the literature on one-part AABs, contended that these materials constitute significant technological progress on the route to commercializing low-carbon cements. They stressed the advantages of this technology over conventional two-part AABs, for it eludes the need for large quantities of corrosive, hazardous activator solutions that entail a health risk and leave a certain carbon footprint.

**FIGURE 10:**
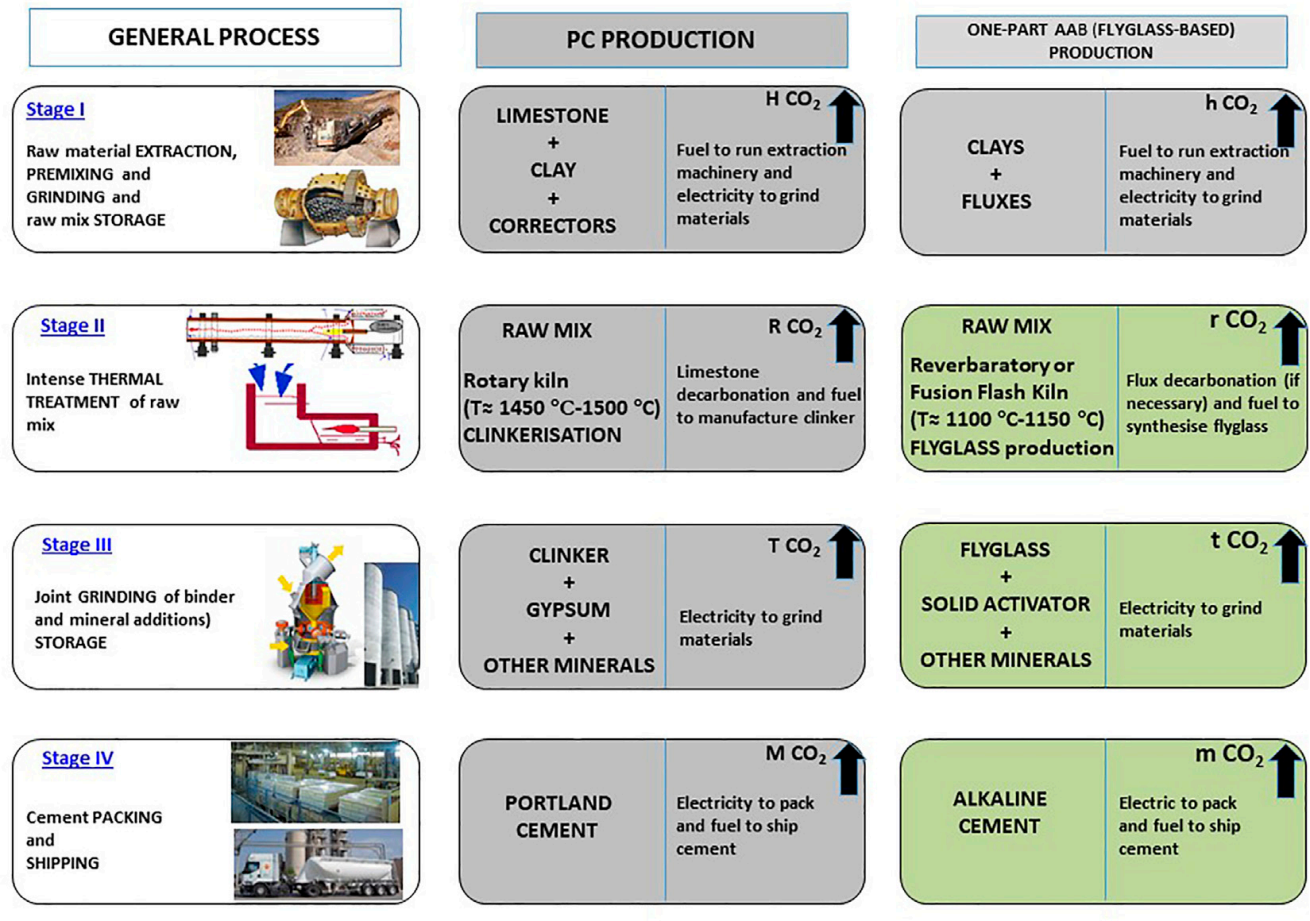
Production and grinding of PC and one-part vitrified clay (flyglass) AAB.

One-part AABs can be prepared with any of the precursors analyzed in sub-section 2.2, although according to the literature blast furnace slag, fly ash and calcined clay are the ones most commonly used (Peng et al., 2017; Hajimohammadi et al., 2018). Similarly, any of the solid activators described in sub-section 2.3 and others not cited hereunder can be used to prepare such cements (Abdollahnejad et al., 2019; Alahrache et al., 2016; Askarian et al., 2018; Askarian et al., 2019; du Toit et al., 2018). From the industrial standpoint, manufacture of these products calls for installing a mill (in the simplest case) or a comprehensive facility similar to the ones in place in PC plants with thermal processing functions for industrial-scale production of the *flyglass* precursor. Two methods can therefore be distinguished for producing one-part AABs, depending on whether the precursor is a blend of non-glassy products or a manufactured glassy product (manufactured like clinker):

1) one-part AAB containing ground fly ash, volcanic ash, slag, calcined clay, etc. (Figure 9A)
2) one-part AAB containing ground *flyglass* (vitrified mineral blend) (Figure 10).

Examples of each process are described below.

#### One-Part Alkali Activated Binders With Ground Not Synthetic Products

Three essential elements must be borne in mind when planning the industrial production of one-part AABs in a mill in which the precursor is an industrial by-product, a natural pozzolan (calcined clay is deemed here to be a natural pozzolan) or a blend of the two.

- The raw materials. A widely range of alkali-activatable industrial waste and by-products as well as a number of highly reactive materials with pozzolanic properties present in nature are presently available for use as raw materials (Duxson et al. 2008; De Rossi et al., 2020; Mobili et al. 2020; Zhou et al., 2020). Small proportions of lime and/or Portland clinker as a source of calcium may also form part of the end binder (Xiang et al., 2018). Possible solid-state activators are described in sub-section 2.3. All raw materials should naturally be available within a reasonable distance of the facility.
- The design. Correctly dosing all the raw materials is vital to optimizing the characteristics of each and ensuring the manufacture of high quality cement with reasonable technical properties (Pavithra et al., 2016; Lahoti et al., 2017; Ning et al., 2019)
- The specific surface of the end product. In this type of facility, grinding has a dual purpose. It serves to uniformly blend all the raw materials used and to afford the end product a suitable specific surface to guarantee particle reactivity with water, factors that differ very little from the requirements in place for PC manufacture. Grinding in an industrial facility obviously plays a critical role in the production stream (the choice of the mill is important) (Mucsia et al., 2015; Dietel et al., 2017; Fernández-Jimnez et al., 2019b).

Descriptions of at least two examples of one-part AABs produced in industrial facilities can be found in the literature: Le Purdociment (Buchwald et al., 2015), and H-cement (Martauz et al., 2016).

***Le Purdociment*** (***clinker-free cement***) ***.*** This AAB, referred to in *Does the Existing Legislation Accommodate Sustainable Binders?* Section and commercialized in Brussels in 1952 by SOFINA, consisted essentially in blast furnace slag and a solid alkaline activator (possibly Na_2_CO_3_). It was used in a number of buildings in Brussels in the nineteen fifties that are still in good service condition, confirming that one-part AABs could be produced industrially (Buchwald et al., 2015)*.*

**H-cement** (**cement with a small fraction of clinker**)**.** In 2012 a Slovakian cement manufacturer (Považská cementáreň, a. s., Ladce) patented a product called H-CEMENT (SK Certificate of Conformity H-CEMENT, 2013), consisting in a blend of essentially 80% alkaline cement (aluminosilicate precursors and solid activator) and 20% clinker. The first industrial-scale pilot tests to produce H-cement began in 2011 at a plant where Považská cementáreň normally manufactures EN 197–1 class CEM I 52.5 R cement. Industrially produced hybrid H-Cement, is characterized by ready grindability, to a specific surface of 6,100 cm^2^/g, an output of 22 t/h and an energy consumption of 63 kWh/t, compared to an output of 12 t/h and energy consumption of 115 kWh/t for conventional CEM I 52.5 R cement with a specific surface of 5,100 cm2/g produced in the same facility (Martauz et al., 2016).

In recent years, much information has been published on the hydration mechanisms in hybrid cements (such a H-cement) and their physical and mechanical properties, along with their durability (Garcia-Lodeiro et al., 2018; Fernández-Jiménez et al., 2019b; Askarian, et al., 2018; Valencia-Saavedra and de Gutiérrez, 2020; Xue et al., 2021).

#### One-Part Alkali Activated Binder Prepared With a Synthesized Precursor

This process exhibits similarities with clinker production and grinding for conversion to PC (Figure 10). Manufacturing AAB with a synthesized precursor will need grinding, thereby adding to the cost and environmental burden but is in step with the foreseeable industrial development of the materials described in sub-section 2.2, which would indisputably ensure the production of universal AABs.

In a parallel vein, ASCEM (Dutch firm) developed, patented and commercialized cements manufactured by alkali-activating synthetic glass, applying production methods (ASCEM, 2020) for over 15 years that yielded a high-strength material with long durability. The company synthesized a calcium aluminosilicate glass by melting a diverse mix of minerals, taking the composition of the vitreous phase of blast furnace slag as a reference. ASCEM’s alkali-activated glass is a high quality cement proven to be apt for structural concrete members (Buchwald, 2012). According to the manufacturers, this cement (with its 85% recycled material, exemplary for its circular economy connotations) emits 50–80% less CO_2_ than PC.

The company tested the cementitious properties of their synthetic glass with traditional two-part alkaline activation. Nonetheless, there is every reason to believe that like Purdociment and H-cement, ASCEM’s precursor glass, or any other with a suitable composition, could be converted to a powder able to react with water if jointly ground with a solid activator. More than that, industrially manufactured glass precursors designed around flyglass composition (similar to type F fly ash with a minimal lime content), might even be deemed the SCM needed to manufacture blended cements and/or traditional concretes in suitable facilities anywhere in the world to replace the dwindling stocks of fly ash (Hisseine and Tagnit-Hamou, 2020; Khan et al., 2020; Kechagia, et al., 2021; Gebremariam et al., 2021).

In short, along similar lines, assuming that construction cements may ultimately become sustainable, serious thought might be given to the idea of inverting the traditional design of blended cements. Whereas a “traditional” blended cement may be defined as bearing clinker (majority component) + gypsum + SCM + limestone, a sustainable alternative might consist in a blend of flyglass (majority component) + alkaline activator + SCM + limestone. In the latter case, the minority clinker and/or lime could be regarded as transitional twenty-first century SCMs.

Figure 10 compares the widely known industrial processes involved in producing PC to those for an alkaline cement manufactured by jointly grinding flyglass with a solid alkaline activator. As the figure shows, manufacturing an alkaline cement from a mix of vitreous precursor + alkaline activator and the respective mineral additions scantly differs from PC manufacture from a mix of clinker + gypsum and the respective mineral additions. That is an important consideration for, among others, most industrial facilities presently producing clinker could manufacture flyglass with no need for major rehaul or investment, which would in any event be justified on sustainability grounds.

## Economic, Legislative and Sustainability Issues

Some brief discussion would appear to be in order around three elements that condition the industrial viability of any new material brought to the construction market: 1) cost-effectiveness, 2) compliance with the existing legislation and 3) sustainability.

The information on alkaline cement/concrete cost-effectiveness published is confined almost exclusively to two-part binders in certain specific regions (Kumar and Kumar, 2014; Oyebisi et al., 2019). Hence it neither can nor should be deemed representative of the broad spectrum of materials and processes apt for AAB production (any more than PC cost-effectiveness should be deemed to be the same in different regions; in fact, at any given time one and the same Portland cement manufacturer may be earning a huge profit in one country while losing money in another). The cost-effectiveness of an AAB plant may consequently be conditioned by factors other than precursor or activator price or even plant operating costs (energy, labour, maintenance, overhead … ), despite the weight of all such costs in companies’ business plans.

One essential variable cannot be ignored when estimating the cost-effectiveness of any binder for use in construction in today’s economic context, however: the price of CO_2_. In January 2021 the subsidies allocated to cement producers in connection with CO_2_ emissions were lowered and now 20% of those emissions will have to be traded on the free CO_2_ market. That will indisputably raise production costs to levels that will preclude many of the export/import transactions presently in place (*Ad Lightart - Global Cement Magazine, March 2020*). In addition, inasmuch as the rules are set by the market, clinker manufacturers with high but not fully used installed capacity tend to over-produce (manufacture more clinker than can be absorbed by the local demand for cement) to lower production costs and dump the local surplus on export markets. Would that practice persist if the price of CO_2_ were to rise substantially? Would it persist if an alternative to Portland cement could be brought to market?

In that vein, the price of CO_2_ will certainly play an essential role in the timing of implementing the changes required in the cement industry. The price of carbon is actually a key and perhaps a decisive climate policy tool to ensure compliance with the Paris Agreement goals. It might not only prompt the industry to lower emissions in the short term, but also spur short-, medium- and long-term innovation. Rather, the price of CO_2_ should be used as a supplementary tool alongside other policy instruments such as public investment. At this time, worldwide initiatives establishing CO_2_ prices apply to around 8 Gt of emissions, or approximately 15% of total greenhouse gases. Current prices are in any event much lower than recommended by climate policy analysts (Boyce, 2018).

In that scenario, some areas of the world have already established penalties for releasing CO_2_ into the atmosphere. Canada, for instance, will raise the price of CO_2_ emissions from the $10 charged in 2018 to $50 in 2022 (Moncef and Yassine, 2020). The conclusion essentially to be drawn is that future AAB cost-effectiveness may very likely depend more on the penalties imposed for emitting CO_2_ (elimination of subsidies) than on the cost of the precursors and activators needed for the manufacture of such binders.

### Does the Existing Legislation Accommodate Sustainable Binders?

Although many young authors still believe (and assert in the literature) that alkaline cements are a recent development, AABs and geopolymers are actually known to have been conceptually fathered over 120 years ago. The earliest known documents with reference to alkaline cement date back to the 19th century (patent No. 544706 titled ‘Manufacture of cement’ by Whiting, 1895), although the Romans, consciously or otherwise, may have deployed alkaline activation in their works (Jackson et al., 2017; Palomo et al., 2019).

The scientific and technical understanding around these materials has naturally progressed enormously since XIX till XXI century. For instance, in 1940 A.O. Purdon contended that “Although slag may be considered to be a cement in itself, hydration proceeds with such extreme slowness that it cannot be used alone as such. A relatively small quantity of an alkali is a much more efficient accelerator” (Purdon, 1940). Why then, nearly a century later, are these materials still only marginally used in construction? Does the answer to that question lie in the technical or economic deficiencies inherent in AABs (alleged lack of raw materials or unsustainable activators) or should it perhaps be sought in the policies in place (standards) in much of the world that protect PC?

Buchwald et al. (2015) published a paper containing much valuable information on Purdon’s initiative to be the first company to commercialize a binder other than PC in the 20th century (in the nineteen fifties Purdon built industrial facilities where he began to manufacture an AAB he called “le Purdociment”). In their paper, Buchwald et al. contend that:

1) In 1956, shortly after Purdociment was launched on the market, the owners of SOFINA (owner of the Purdociment cement plant) received an offer from the association of Belgian cement manufacturers to cease Purdociment production.
2) No information has been found on Purdociment output (the documents disappeared some time in the past).
3) The Purdociment consisted in a blend of blast furnace slag and alkaline salts.

Attention should be drawn here to the parallel timing in the nineteen fifties between the enactment in Western Europe of the earliest standards on PC composition and the disappearance of any alternative cementitious material that, like Purdociment, might be deemed apt to compete PC on the construction materials marketplace. Those first Western European standards not only established compositional limits for construction cements, but required the use of clinker as an irreplaceable component of those materials.

Whereas the protectionist standards in place in free Europe banned the commercialization of binders other than PC, the Soviet Union opened its gates to the use of AABs (historic paradox). In the second half of the 20th century, the USSR published over 60 standards and specification on AABs (Shi et al., 2003), and the many buildings and infrastructures built in Russian and Ukrainian cities in the nineteen fifties (Berg et al., 1970), still in service today (Shi et al., 2003), confirm not only the suitability of those materials (endorsed by ad hoc local legislation) but the existence of plants producing them.

Today a series of rules and regulations on cements and concretes for construction on the books in the vast majority of countries prohibit the use of materials that do not bear a certain percentage of clinker (absurdly, in twenty-first century it would be forbidden to build Agrippa’s pantheon the way it was constructed by the Romans 2000 years ago). At the same time some authors rightly question the present legislation that ensures the quality of concretes made with PC (Douglas Hooton, 2015; Douglas Hooton, 2019; Vanderley et al., 2019) against a backdrop of legislation apparently favourable to construction binder ‘unsustainability’. In short, the time is ripe to acknowledge the mitigating effect that the immediate use of AAB in construction would have on the global environmental impact of cements and concretes and to foster the universal application of AABs with the publication of worldwide, inclusive standards geared to fostering the use of eco-friendly binders. The rules presently protecting PC should be thoroughly revised in the very short term (it took CEN 30 years to adopt Europe-wide cement standard EN 197) (Sanjuán and Chinchón, 2014) to establish a new legislative ethic (perhaps based on cement performance, as in ASTM standard C1157) consistent with the environmental need to lower the cement and concrete carbon footprint. The absence of political initiatives favouring the industrial development of new cementitious materials is no longer justified. Against that backdrop, the technical, economic, safety and other arguments wielded to date for banning AABs in construction are no longer valid.

### Sustainablility: Driving Force Behind AAB Development

Sustainability is associated with the limits that should govern generational legacies and consequently it is not an option but a challenge faced by today’s generations to ensure our individual and collective life projects do not compromise the capacity of generations to come to meet their needs. Sustainability is related to the planet’s capacity to satisfy the needs of its inhabitants (Rao, 2000).

The literature analyzing the environmental impact of construction materials, while still insufficient, has often revealed that compliance with existing worldwide environmental recommendations will entail lowering the CO_2_ emissions attributed to cement production by a “factor of 4” by 2050 (Komnitsas, 2011). According to the most optimistic estimates, however, the abatement strategy endorsed by cement industry may suffice to design cements able to shrink the carbon footprint by a “factor of 2”, but not of 4 (IEA, 2017). The latter would require a much less conservationist stance—essentially a clean break - than exhibited by those with the responsibility to decide the impact of cement on future generations.

In this universal dilemma, as alkaline cements hold great technical potential, broadly supported by scientific and technical evidence, they may constitute the key to producing low environmental impact concrete in the immediate future; although much good quality research will be needed in the near future in order to clarify the so many discrepancies existing in the scientific literature related to the carbon footprint of AABs. Actually, the sustainability of alkaline concrete production has been the object of research for over a decade. Most research on AAB sustainability has been based on LCA (life cycle assessment) and similar procedures for assessing the environmental impact of production. Some authors (Robayo-Salazar et al., 2018) adopt the “cradle to gate” criterion, which includes raw material extraction, transport to the production site and any on-site GHG emissions resulting from raw material conversion. In other words, “cradle to gate” (unlike “cradle to grave”) fails to take the post-construction environmental impact of materials into consideration. Nonetheless, that practical criterion should be deemed sufficiently valid for comparing the AAB and OPC carbon footprints. Many papers have now been published attesting to the status of AABs as “sustainable materials” (Turner and Collins, 2013; Adesina, 2020; Huseien and Shah, 2020).

Weil et al. (2009) pioneered life-cycle assessment of the environmental impact of two-part alkaline concrete. Later Habert et al. (2011) studied mixes similar to those used by Weil et al., (Weil et al., 2009) concluding that geopolymer two-part concretes have a lower impact on global warming than attributed to Portland concrete, while also stressing the substantial environmental footprint associated with the liquid activators (sodium silicate in particular) used to manufacture AAB concrete. It was in that context that Habert et al. (2011) noted that two-part alkaline concretes manufactured with fly ash lowered CO_2_ emissions by 45% relative to a standard PC concrete mix. Later on, the same author (Habert and Ouellet-Plamondon, 2016) revealed the discrepancies in the literature around calculations of the GWP attributed to two-part AAB mixes. Although GWP is lower in fly ash than in slag, since in concretes with similar mechanical strength higher doses of activator are needed for the former, GWP does not differ significantly between the two materials. The inference is that the three examples cited at *Raw Material Processing. Industrial-Scale Alkali Activated Binders Manufacture* section (Australian airport, motorway near London and Vertua concrete) may have reduced GHG emissions to a similar degree.

To the extent that the liquid activator used (particularly sodium silicate) determines two-part AAB sustainability (Fawer et al., 1999; Mellado, et al., 2014) Habert advocated for more detailed study on lowering the dose of traditional activators or the use of alternative activators with no or only a small environmental footprint.

From the standpoint of sustainability of one-part AAB similar to Purdocement or the H-cement described in *Raw Material Processing. Industrial-Scale Alkali Activated Binders Manufacture* Section, the authors of the present article deem that this option should be regarded as a highly effective alternative in terms of reducing GHG emissions. Habert and Ouellet-Plamondon (2016) assessed the environmental impact of a number of alternative one- and two-part AABs and hybrid cement. They concluded that hybrid cements (similar to the aforementioned H-cement), in addition to exhibiting a GWP (global warming potential) 70% lower than PC, constitute a promising marketable material for transitioning from new Portland-based formulations such as LC3 cements, expected to deliver 40% abatement (Martirena and Scrivener, 2017; Scrivener K. et al., 2018; Scrivener K. L. et al., 2018), to clinker-free AABs. The authors further concluded that despite the uncertainties around the accuracy of the environmental impact of alkaline concretes, they may ultimately be decisive in lowering the cement industry’s CO_2_ emissions. The study by Habert and Ouellet-Plamondon (2016) addressed only the environmental impact associated with AAB production (cradle-to-gate embodied carbon), however, omitting (once again) any assessment of the material’s durability (cradle-to-grave embodied carbon).

Be it said that no data have been published to date on the environmental impact of AABs processed with an industrially manufactured precursor such as described in Figure 10. That does not preclude a brief qualitative analysis of the key features characterizing their carbon footprint relative to PC, however (quantitative assessment would require specific studies), or concluding that the manufacture of sustainable cement for construction is feasible.

Hence, stage I in Figure 10 (raw material extraction, premixing and grinding) differs in the Portland and one-part AAB cement processes, for quarrying and grinding limestone is not exactly the same as collecting and grinding clay and other aluminosilicate materials, although such differences, expressed in terms of CO_2_ emissions (‘H’ vs ‘h’ in Figure 10) would have no significant effect on the cement industry’s overall emissions.

The emissions associated with Stage IV (‘M’ vs ‘m’) may also be assumed to be equivalent in the two processes. Some thought is also be given, however, to the shipment (export/import) of large volumes of PC that today increase its embodied CO_2_ by 5% (Global Cement Magazine, March 2020). The key question is: might the presence on the marketplace (with widespread industry acceptance) of an alternative, competitive and versatility manufactured binder lower cement imports/exports and consequently global CO_2_ emissions?

The key to the environmental impact of any PC, however, lies in Stage II, clinkerization, and the CO_2_ emissions induced by fuel combustion and limestone decarbonation (Richardson and Taylor, 2018). Although the thermal stage (an intense process) of production is likewise important in one-part AAB, it involves clearly lower emissions than PC manufacture given the lower kiln temperature (∼1,150°C for flyglass compared to 1,450°C for clinker) and especially the near absence of carbonate in the materials, primarily clay, used to make flyglass. ‘R’ in Figure 10 would obviously appear to be considerably greater than ‘r’.

In Stage III (Figure 10), the embodied CO_2_ would be greater in one-part AAB than in PC (‘t’>’T’) when the alkaline cement bears a certain amount (∼20%) of clinker or lime. That component, however, might not necessarily be required in all circumstances, in which case ‘T’ and ‘t’ would be similarly intense (we are assuming the solid activator to be a natural product).

Indisputably, accurate assessment of the potential sustainability of alkaline cements/concretes, particularly of one-part AABs based on LCA or similar calculations of their environmental impact, is imperative. Nonetheless, acknowledging the need to enhance the understanding and optimize the practical application of AABs should not be wielded as a reason for stalling their technological development. Rather, the sooner that challenge is tackled, the sooner will the environmental issues associated with the construction materials industry be solved.

The vast majority of the papers published accord AABs excellent durability, a key issue in rigorous LCA. Although a detailed description of the durability of alkaline concretes (be they one- or two-part AABs) lies outside the scope of this article, for such a review would call for another paper of similar bibliographic amplitude, that property cannot be overlooked when pursuing maximum accuracy in environmental impact assessments. Further information on the durability of these materials can be found in the following references (Adam, 2009; Fernandez-Jimenez et al., 2007a; Fernández-Jiménez and Palomo, 2009; Kupwade-Patil and Allouche, 2012; Provis et al., 2014b; Miranda et al., 2005; Hossain et al., 2015; Arbi et al., 2016; Bačuvčík et al., 2017; Saravanakumar and Kalaivani, 2020; Wang et al., 2020; Tian Lingyu et al., 2021).

In short, economy and sustainability (two concepts nowadays playing a key role to materialize the industrialization and commercialization of any binder for the construction sector) seem to play in favor of alkaline cements when compared with PC. Regarding regulations, it can only be said that most of the standards in the world are demanding the use of large amounts of clinker in cements and therefore are incompatible with the need of sustainable framework for regulations.

## Concluding Remarks

Stricter international measures to tackle climate change than presently in place would seem to be needed in the immediate future if carbon neutrality is to be attained by 2050. That will affect the cement industry, which continues to deem PC the sole possible alternative for the future of construction, despite its enormous environmental impact. This article wields a series of arguments to counter opinions that underestimate or even deny the viability of alkaline cements as a solvent alternative for the building industry, even if, technologically speaking, alkaline cements and “portland-alkaline” hybrid cements have proven to sufficiently meet the physical-mechanical demands (compressive strengths at 2 and 28 days, setting times … ) which today, through the standards, are required of binders for its use in the construction sector. For example, and to mention just a few cases:

**✓** Alkaline activation (with salts such as carbonates, sulphates, silicates, etc.) of mixtures of blast furnace slag (AAS), fly ash and small proportions of clinker, at room temperature, can achieve compressive strengths of more than 40 MPa (more than enough for most of the applications in architecture and civil engineering) (Donatello et al., 2013; Garcia-Lodeiro et al., 2013a; Martauz et al., 2016; du Toit et al., 2018).
✓ For synthetic glasses, depending on the CaO content and the activation conditions, it is possible to generate materials with compressive strengths of between 10 and 60 MPa (Buchwald, 2012; Garcia-Lodeiro et al., 2016a; Ruiz-Santaquiteria et al., 2016).
✓ For preursors with low calcium contents such as type–F fly ash. metakaolin or other calcined clays, high alkaline concentrations., as well as curing temperatures above 65°C are currently needed (especially interesting in the case of pre-cast elements) (Fernández-Jiménez et al., 2005a; Duxson et al., 2007a; Buchwald et al., 2009; Ruiz-Santaquiteria et al., 2013b). In these cases, is possible to design materials with compressive strengths of up to 30 MPa at 28 days, and even higher than 60 MPa, if the particle size is strictly controlled (Fernández-Jiménez et al., 2003b and 2007b).

Alkaline cements and Alkaline Hybrid cements, in terms of durability (Adam, 2009; Law et al., 2015, Bačuvčík, et al., 2017; Wang et al., 2017, 2020) show, in most of cases, a similar behavior to PC, although it is true that they stand out for their excellent behavior against acid attack and for their extraordinary resistance to fire (Donatello et al., 2014).

However, for the implementation of these materials it is necessary:

1) Encourage policies to further the use of new cements (by making policy-makers aware that a substantial percentage of the scientific community firmly believes cements other than the Portland variety are feasible in the immediate future)
2) Introduce the use of new cements in international standards (by identifying the pressing need to draft international standards clearly geared to sustainability and endorsement of the use of other than Portland-type cements)
3) Refute the technological bias in the alleged lack of raw materials (by processing raw materials in abundant supply other than limestone with scant embodied CO_2_ is a fast-track approach to producing uniform, high quality cements anywhere in the world)
4) Parry the objections around the limitations to the use of alkaline products (by raising awareness of the possibility of generating high pH *in situ* via chemical reactions between (abundant) neutral alkaline salts and (likewise copious) alkaline-earth salts)
5) Highlight the true sustainability potential in AABs (prioritizing sustainability in cements intended for construction is the sole argument relevant to this debate).

## Authors’ Note

This article was drafted during lockdown in the struggle against the expansion of covid- 19. Hosts of scientific articles have related climate change and its implications to biodiversity, not just human life. The corona virus crisis has taught us that: 1) we cannot afford longer attacks against this planet (our mutual home); and 2) when the international scientific community joins forces to solve a problem, solutions are feasible.
